# IMplementation of Physical Activity for Children and adolescents on Treatment (IMPACT) for Cancer Diagnoses in Alberta: Protocol for a Single-Arm, Mixed-Methods, Hybrid Effectiveness-Implementation Trial

**DOI:** 10.2196/59302

**Published:** 2025-12-17

**Authors:** Emma McLaughlin, S Nicole Culos-Reed, Carolina Chamorro-Viña, Beverly Wilson, Sara Fisher, Gregory MT Guilcher, Bridget Penney, Mira Penney, Laura Wich, Janine Wich, Amanda Wurz

**Affiliations:** 1 Faculty of Kinesiology University of Calgary Calgary, AB Canada; 2 Department of Oncology, Cumming School of Medicine University of Calgary Calgary, AB Canada; 3 Department of Psychosocial Resources, Arthur JE Child Comprehensive Centre Alberta Health Services Calgary, AB Canada; 4 Kids Cancer Care Foundation of Alberta Calgary, AB Canada; 5 Department of Pediatrics University of Alberta Edmonton, AB Canada; 6 Stollery Children's Hospital Edmonton, AB Canada; 7 Hematology/Oncology/BMT Clinic Alberta Children's Hospital Calgary, AB Canada; 8 Participant Advisory Board Calgary, AB Canada; 9 School of Kinesiology University of the Fraser Valley Chilliwack, BC Canada; 10 Research Institute BC Children’s Hospital Vancouver, BC Canada

**Keywords:** exercise, movement, pediatric oncology, neoplasms, internet-based, online, remote

## Abstract

**Background:**

Physical activity (PA) is feasible, safe, beneficial, and recommended for pediatric patients with cancer. Nevertheless, PA levels remain low due to treatment-related effects (eg, fatigue, pain), isolation and immunosuppression, geographic and transportation barriers, and limited access to population-specific programming. Delivering PA by videoconference may address some of these barriers. Thus, IMplementation of Physical Activity for Children and adolescents on Treatment (IMPACT) was developed. IMPACT is an individualized, tailored PA intervention delivered by an exercise professional over videoconference and is being evaluated in a hybrid effectiveness-implementation trial.

**Objective:**

The objectives of this trial include (1) assessing the effectiveness of IMPACT on participants’ device-measured PA (primary effectiveness outcome) and secondary effectiveness outcomes, including participant- and caregiver-reported PA and participant- and caregiver-reported quality of life, symptoms, cognitive function, resource use, and participant physical fitness outcomes (ie, participants’ aerobic endurance, lower body flexibility, shoulder flexion range of motion, balance, functional mobility); and (2) assessing the implementation of IMPACT and the trial through evaluating recruitment, indices of feasibility, delivery time, expertise, cost, fidelity of intervention delivery, and adverse events.

**Methods:**

A single-arm, mixed-methods, type II hybrid effectiveness-implementation trial is being conducted, and the RE-AIM (reach, effectiveness, adoption, implementation, maintenance) framework is guiding evaluation. Pediatric patients with cancer and blood disorders diagnosed between 5 and 18 years of age are being recruited in Alberta. Following informed consent/assent and baseline assessments (ie, participants’ device-measured PA, participant- and caregiver-reported outcomes, participant physical fitness outcomes), participants receive individualized, tailored PA sessions up to 3 times/week for 15-45 minutes/session over 12 weeks from an exercise professional via videoconference. Assessments are then completed postintervention (with the addition of a participant and caregiver interview) and at 6- and 12-month follow-ups. With respect to RE-AIM, reach covers referral and participation rates, participants’ and caregivers’ personal and medical information, and the proportion of participants from rural versus urban locations. Effectiveness includes participants’ device-measured PA, participant- and caregiver-reported outcomes, and participant physical fitness outcomes. Adoption covers sources of referral. Implementation includes recruitment, feasibility, delivery time, expertise, cost, fidelity of intervention delivery, and adverse events. Maintenance includes participants’ and caregivers’ desire for ongoing access to PA, and participant- and caregiver-reported PA levels at 6- and 12-month follow-ups. Throughout the trial, quality improvement cycles occur every 6 months.

**Results:**

Funding was obtained in April 2022. Participant recruitment began in March 2022 and will conclude in December 2025. Interim analyses and additional analyses of selected trial data have been conducted, and publications are forthcoming. Full trial results will be analyzed and published following trial cessation.

**Conclusions:**

This work will provide insights into the effects of, and factors influencing, the implementation of an individualized, tailored PA intervention delivered by videoconference. Findings may support continued exploration of videoconference delivery as a strategy to ensure that more pediatric patients can access and benefit from PA.

**Trial Registration:**

ClinicalTrials.gov NCT04956133; https://clinicaltrials.gov/ct2/show/NCT04956133

**International Registered Report Identifier (IRRID):**

DERR1-10.2196/59302

## Introduction

### Background

Cancer among children and adolescents (ie, cancers diagnosed at <18 years) is relatively rare [1]. Recent estimates suggest that approximately 400,000 children and adolescents are diagnosed with cancer each year globally [2], representing <3% of all new cancer diagnoses annually [3]. Nearly 90% of children and adolescents diagnosed with cancer are anticipated to survive 5 or more years, and >70% will survive 10 or more years [4]. Despite this relatively positive prognosis, the disease remains a leading cause of morbidity and mortality among children and adolescents in North America [1,5], and is associated with negative effects in both the short term [6-8] and long term [9-14].

In the short term, children and adolescents on treatment for cancer (ie, pediatric patients with cancer) experience negative effects spanning physical (eg, pain, nausea [6]), psychological (eg, depression, anxiety [7]), social (eg, isolation [7]), and cognitive (eg, difficulty thinking and remembering [8]) domains of functioning. In the long term, children and adolescents living beyond treatment for cancer (ie, pediatric cancer survivors) may experience poorer physical (eg, decreased strength, increased fatigue [14]), psychological (eg, posttraumatic stress, fear of cancer reoccurrence [10,14]), social (eg, challenges in relationships with family and friends [10]), and cognitive (eg, reduced processing speed, impaired executive functioning, lower academic performance [9,15]) outcomes. This cohort is also at greater risk for secondary cancers, morbidity, and premature mortality [16-20]. Thus, identifying strategies to improve outcomes in both the short and long term is important.

Physical activity (PA; any bodily movement produced by skeletal muscles that requires energy expenditure [21]) may enhance physical, psychological, social, and cognitive domains of functioning among pediatric patients with cancer [22-24]. PA may reduce the severity of many of the negative effects experienced during treatment and enhance the overall quality of life among pediatric patients with cancer [25-27]. Indeed, systematic reviews have found that individualized, supervised PA during treatment—even during the intensive treatment phase—is safe and may improve a range of health outcomes [28,29]. Further, PA during treatment is associated with reductions in morbidity, secondary cancers, and premature mortality [30]. Recent reviews underscore the beneficial role of PA for pediatric patients with cancer [28,29,31,32], and guidelines and recommendation statements have been published suggesting that all pediatric patients with cancer *move more* [33].

Despite the documented benefits of PA and existing guidelines and recommendation statements [33], the desire and motivation of pediatric patients with cancer to engage in PA varies widely [34], and many stop engaging in PA during treatment [35,36] or take part in significantly less PA than their healthy peers [37]. This may be due to many of the barriers pediatric patients with cancer face when seeking to engage in PA, including adverse treatment-related side effects (eg, pain, nausea, fatigue), variable treatment timelines that differ significantly from child to child and include periods of isolation and immunosuppression [38,39], and the lack of pediatric cancer-specific PA opportunities (eg, interventions or programs) for this group [40]. Of the PA interventions and programs that are available, most are delivered at or near the hospital, which could pose an additional barrier to participation [41,42] and further disadvantage those who live in rural locations or farther from the hospital. Additionally, given that pediatric patients with cancer and their caregivers desire PA to be delivered close to or at home, the dominant focus on hospital-based delivery overlooks important preferences for PA within this population [41,42].

Exploring home-based PA delivery may be one way to address some of the barriers pediatric patients with cancer face when trying to engage in PA. However, relying solely on home-based PA programs may not successfully promote the adherence required to realize PA-related outcomes among this group [43]. Synchronous supervision of PA sessions by videoconference from exercise professionals may better support adherence to PA [40,43], ensure safe PA engagement, and reach more pediatric patients with cancer—a small and dispersed population [44]. Emerging evidence from recent trials suggests PA delivered by exercise professionals over videoconference is feasible and may improve physical outcomes (eg, physical fitness, flexibility, strength) among pediatric cancer survivors (ie, those off-treatment) [45,46]. This is bolstered by findings from videoconference-delivered (ie, telehealth) interventions focused on rehabilitation for pediatric patients with cancer and pediatric cancer survivors, which have found significant improvements in outcomes spanning PA limitations, participation restrictions, and quality of life [47,48]. In addition to being feasible and conferring benefits, videoconference delivery of PA may address another important interrupter to supervised in-person PA delivery—namely, circumstances such as cold and flu season or pandemics that limit access to pediatric patients with cancer (eg, ward closures) [45-48]. Finally, videoconference delivery of PA to this cohort could ensure that more pediatric patients with cancer have access to support from trained exercise professionals (eg, kinesiologists, clinical exercise physiologists, certified personal trainers), filling a gap within the Canadian context where exercise professionals (beyond physiotherapists and occupational therapists) are rarely, if ever, embedded within the pediatric cancer care system.

Indeed, in Alberta, there are no exercise professionals embedded within the pediatric cancer care system. However, there is support for providing PA in this cohort, positioning supervised videoconference PA delivery as a viable modality. Considering a desire to implement and evaluate a PA intervention with potential for sustainability and scalability in this context, a single-arm, type II hybrid effectiveness-implementation trial [49] is ideally suited to generate data assessing both the impacts of PA delivered by videoconference and the factors influencing implementation. This trial design has also been recommended by international experts to advance the field of pediatric exercise oncology [50]. Though videoconference delivery is a relatively novel approach, forgoing traditional feasibility and comparative designs is suitable given: (1) face validity for PA and its acceptability for pediatric patients with cancer [31,51]; (2) a growing evidence base from different, but related, populations (eg, young adults affected by cancer, adolescents with intellectual and developmental disabilities, adolescents with congenital heart disease) supporting the delivery of PA by videoconference [52-55]; (3) minimal risk associated with individualized, tailored PA for pediatric patients with cancer—with evidence to date supporting the safety of PA during this phase and of videoconference delivery (ie, few adverse events) [24,28,29,56]; and (4) rationale to gather more data on the effectiveness of PA interventions for pediatric patients with cancer [50] and on implementation metrics [47,48].

### Purpose and Study Objectives

The co-primary aims of this trial are to (1) assess the effectiveness of the videoconference-delivered PA intervention on participants’ device-measured PA (primary effectiveness outcome), as well as secondary effectiveness outcomes including participant- and caregiver-reported PA and participants’ quality of life, symptoms, cognitive function, resource use, and physical fitness outcomes (ie, aerobic endurance, lower body flexibility, shoulder flexion range of motion, balance, functional mobility); and (2) assess implementation of the PA intervention and the trial through evaluating recruitment, indices of feasibility, delivery time, expertise, cost, fidelity of intervention delivery, and adverse events.

## Methods

### Overview

This protocol paper was prepared following the SPIRIT (Standard Protocol Items: Recommendations for Interventional Trials) guidelines [57] and the TIDieR (Template for Intervention Description and Replication) checklist [58] (see Multimedia Appendix 1). The trial was registered with ClinicalTrials.gov [59] on July 09, 2021, and started at Stollery Children’s Hospital on March 1, 2022, and at Alberta Children’s Hospital on September 28, 2022 (see the “Ethical Considerations” section). This paper details the PA intervention and trial to enhance transparency in reporting and will serve as a reference for forthcoming papers. The CONSORT (Consolidated Standards of Reporting Trials) for eHealth interventions [60], the StaRI (Standard for Reporting Implementation Studies) statement [61], and the GRAMMS (Good Reporting of a Mixed Methods Study) checklist [62] will be followed when preparing and reporting the full trial results.

### Trial Design

A single-arm, mixed-methods, type II hybrid effectiveness-implementation trial is being conducted, and the RE-AIM (reach, effectiveness, adoption, implementation, maintenance [63,64]) framework is being used to guide evaluation. As above, a single-arm trial design was selected with consideration for the local Alberta context and the desire to develop and test an intervention that could be sustained and scaled into a routine care pathway. Mixed methods are being used to gather both qualitative and quantitative data to provide a more comprehensive understanding of the effectiveness and implementation of this novel mode of delivery. Quantitative and qualitative data are collected from participants, caregivers, and those involved in trial conduct (ie, health care providers, exercise professionals) at several time points, outlined in greater detail throughout the “Procedures” and “Data Collection” sections.

### Ethical Considerations

The research ethics application for this trial was submitted to the Health Research Ethics Board of Alberta (HREBA) on December 16, 2020, and approval was granted on February 16, 2021 (HREBA.CC-20-0364). Administrative approval was obtained from Stollery Children’s Hospital on February 12, 2021, and from Alberta Children’s Hospital on May 15, 2021. Due to delays related to the COVID-19 pandemic and hospital restrictions, recruitment opened at Stollery Children’s Hospital on March 1, 2022, and at Alberta Children’s Hospital on September 28, 2022. The first health care provider referral was received from Stollery Children’s Hospital on March 16, 2022, and from Alberta Children’s Hospital on October 19, 2022. Finally, research ethics board approval was granted by the Human Research Ethics Board at the University of the Fraser Valley on January 23, 2023 (approval number 101287). This approval was sought as the co-principal investigator (AW) moved institutions and onboarded additional local trial team members to support interview conduct and quality improvement cycle data collection.

In accordance with the principles of the Declaration of Helsinki and the ethics boards named above, all patients provide informed consent or assent to participate in this trial online via the web-based platform Research Electronic Capture (REDCap; Vanderbilt University) [65,66]. The designation of a Mature Minor, endorsed by Alberta Health Services, is being followed (ie, typically minors 14 years and older who can understand and appreciate the nature, risks, and consequences of participating in proposed procedures and are able to provide consent without the input of their legal guardian [67]). Caregivers also provide informed consent and, where indicated, provide consent on behalf of their child via REDCap. Health care providers and exercise professionals provide informed consent via REDCap to participate in semistructured interviews at regular intervals (see the “Quality Improvement Cycles” section).

Of note, all trial data are being deidentified and are accessible only to members of the trial team directly involved in trial conduct. Financial compensation is not provided to participants, caregivers, health care providers, or collaborators referring to the trial. Exercise professionals involved in PA sessions and assessment delivery are compensated for their time delivering PA sessions and conducting assessments of physical function, but do not receive compensation for completing interviews or data collection as part of the quality improvement cycles.

### Recruitment and Participants

Patients are recruited through referral from health care providers at Alberta Children’s Hospital or Stollery Children’s Hospital, via consent-to-contact forms, posters, self-referral documents, emails from the Kids Cancer Care Foundation (a local support organization), and word of mouth. Children and adolescents are eligible to participate if they (1) are between 5 and 18 years of age at enrollment; (2) have been diagnosed with any cancer or blood disorders; (3) are scheduled to receive, are currently receiving, or have completed treatment within the past 3 months; (4) are medically cleared to participate in PA; (5) have a caregiver (ie, an individual responsible for providing care to a child or adolescent diagnosed with cancer or a blood disorder) willing to complete assessments and be present during the PA sessions; and (6) are able to participate in English.

The decision to include pediatric patients with blood disorders was made considering that, in this Canadian context (ie, Alberta), pediatric patients with cancer and blood disorders are typically treated on the same unit/ward; the supervised, tailored, and low-risk nature of the PA delivered in IMplementation of Physical Activity for Children and adolescents on Treatment (IMPACT); early evidence suggesting the safety and benefits of PA in this cohort (ie, pediatric patients with blood disorders [68]); and consultations with clinical trial team members who supported, including pediatric patients with blood disorders.

### Sample Size

The trial sample size was set at 76. This estimate was based on the primary effectiveness outcome of device-measured PA (ie, steps per day over 7 days). A meaningful difference in PA was defined as an average increase of 1000 steps/day between baseline (week 0) and postintervention (week 12), based on prior literature [69]. Setting *α*=.05 and power=0.80, and accounting for 25% attrition, a target of 76 participants between the ages of 5 and 18 years, along with one of their caregivers, from Alberta Children’s Hospital or Stollery Children’s Hospital (approximately 38 per site) was established. This sample size also provides sufficient power to explore secondary effectiveness outcomes while accounting for covariates (eg, sex, gender, and other factors) and offers the opportunity to stratify the sample to examine changes by cancer versus blood disorder diagnoses and across age ranges.

Regarding qualitative data, all participants and caregivers are invited and encouraged to complete an interview at postintervention (week 12) to capture their experiences and perspectives. This approach was chosen to gain a comprehensive understanding of participants’ and caregivers’ experiences within IMPACT. Perspectives from those involved in implementation are also being collected. In these cases, health care providers who refer participants and exercise professionals involved in intervention and trial conduct are recruited purposefully, with no predetermined sample size. Instead, the principle of theoretical saturation is applied, wherein participants are recruited until no new insights emerge or specific quality improvement cycle questions have been addressed [70].

### Procedures

**Figure 1:**
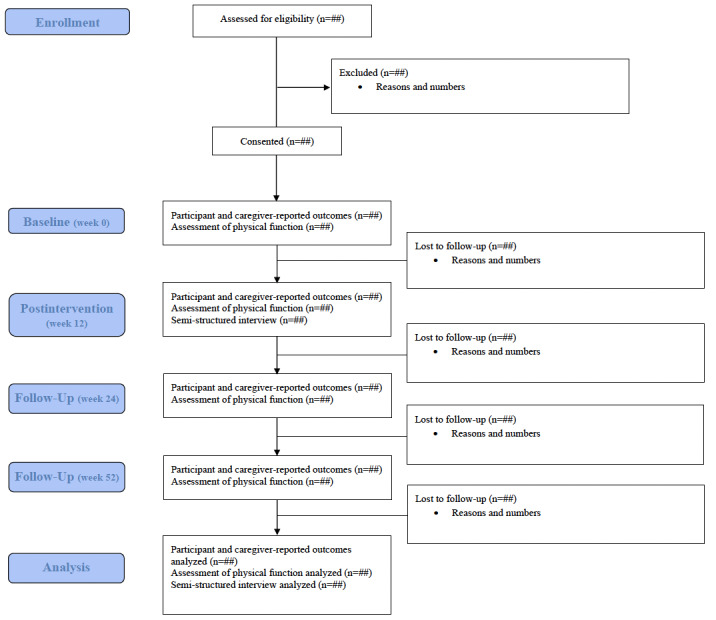
CONSORT flowchart.

Following referral (health care provider or self-referral), a member of the trial team contacts the caregiver via their preferred method (ie, email, phone call, or text message) to describe the PA intervention and trial, ascertain interest, confirm eligibility, and share the link to the participant consent or assent, and caregiver consent forms via REDCap. Once consent (or assent, as appropriate) is provided by the patient (henceforth referred to as the participant) and caregiver, the participant is scheduled for a baseline (week 0) assessment, which includes the collection of the participant’s device-measured PA through a 7-day Fitbit (Google LLC) wear period and tracking log. Caregivers also complete a questionnaire covering personal and medical information, and participants and caregivers complete a series of questionnaires via REDCap assessing participant- and caregiver-reported PA as well as the participant’s quality of life, symptoms, cognition, and resource use. Participants additionally complete a physical assessment via videoconference, measuring aerobic endurance, lower body flexibility, shoulder flexion range of motion, balance, and functional mobility. Once baseline assessments are completed, participants are matched with an exercise professional and begin the 12-week, individualized, 1:1 PA intervention. Following the PA intervention, participants and caregivers complete the same assessments as at baseline (with the exception of personal and medical information, which is collected only at baseline) and are invited to complete a postintervention interview via videoconference. At 6- (week 24) and 12-month (week 52) follow-up time points, participants complete the same assessments as at baseline (week 0) and postintervention (week 12), with the exceptions of personal and medical information, which is collected only at baseline, and the interview, which is conducted only postintervention. To enhance data completeness, notifications of assessments are sent via REDCap, and reminders are sent every 3 days for up to 2 weeks if assessments are not completed at baseline, postintervention, and follow-up time points. See Figure 1 for the CONSORT diagram.

Throughout the trial, additional data aligned with the RE-AIM framework are being collected. Specifically, *reach* (ie, referral rate, number of participants excluded and reasons for exclusion, participation rate, participant and caregiver demographics, and medical information); *effectiveness* (ie, participants’ and caregivers’ perceptions of the IMPACT intervention and trial); *adoption* (ie, sources of referral and differences in referrals across hospitals); *implementation* (ie, indices of feasibility [trial retention, adherence, percentage of missing data, and participants’ and caregivers’ perceptions of feasibility], intervention delivery time, trial delivery time, expertise, cost, fidelity of intervention delivery, and adverse events); and *maintenance* (ie, semistructured interviews with participants and caregivers to explore their desire for ongoing access to the PA intervention, and the proportion of participants who continue to engage in PA through the 2 follow-ups). All data are collected by trial staff. Finally, quality improvement cycles to review and optimize the implementation of the intervention and trial are conducted every 6 months (see the “Quality Improvement Cycles” section). Data covering participants’ and caregivers’ personal and medical information, intervention fidelity, and perspectives from health care providers and exercise professionals involved in referral and trial conduct are collected. These data are collated and reviewed during meetings with an advisory board comprising participants, caregivers, trial staff, health care providers, and community partners (see the “Participant and Caregiver Involvement” section). Further information on data collection and quality improvement cycles is provided in the relevant subsections within the “Data Collection” and “Quality Improvement Cycles” sections, respectively.

### The Intervention

The PA intervention was developed over 24 months with funding support from the Strategy for Patient-Oriented Research Evidence Alliance [71] and followed a patient-oriented approach [72]. The intervention was informed by relevant literature [28,29,31,32], existing pediatric exercise oncology resources (eg, the Pediatric Cancer Patients and Survivors Engaging in Exercise for Recovery program [73,74] and the Pediatric Oncology Exercise Manual [75]), and recently developed PA guidelines and recommendation statements for pediatric patients with cancer and pediatric cancer survivors [33]. This was integrated with perspectives gathered through semistructured interviews with 11 international experts who had successfully implemented PA with pediatric patients with cancer [69,76] and 16 local health care providers (eg, oncologists, nurses, physiotherapists, child life specialists) and staff from children’s hospitals in Alberta. Semistructured interview guides were developed with support from an implementation science expert (Kelly Mrklas) and were informed by the Consolidated Framework for Implementation Research and the Theoretical Domains Framework [77]. Questions explored barriers and enablers to implementing PA with pediatric patients with cancer and examined factors spanning intervention characteristics, outer setting, inner setting, characteristics of individuals, and process. Additionally, during the interviews, context-specific implementation strategies were explored. Relevant literature, resources, and perspectives were integrated, and a team of researchers, exercise professionals, and health care providers (ie, the trial team) collaboratively developed and refined the PA intervention.

The resulting 12-week PA intervention, delivered via videoconference, includes sessions offered 3 times per week for 15-45 minutes and incorporates a combination of aerobic, resistance, balance, core, and flexibility exercises. Videoconference delivery allows participants to join whether they are at home or in the hospital. The intervention is tailored for each participant based on the frequency (ie, how often each exercise is completed and the number of PA sessions per week), intensity (ie, the difficulty of exercises or PA sessions), time (ie, duration of each exercise and each PA session), and type of exercise (ie, aerobic, resistance, balance, core, and flexibility). Each aspect of the intervention is modified based on participants’ needs, preferences, medical factors, age, and abilities, including how they are feeling and managing treatment-related side effects, their treatment schedules, and other considerations (eg, school or extracurricular commitments). PA sessions are provided regardless of whether participants are in the hospital or at home. Select behavior change techniques (eg, goal setting, feedback and monitoring, social support, self-monitoring) are embedded within PA sessions and discussed in an age-appropriate manner. Behavior change topics are also selected based on participant choice and ongoing conversations throughout the PA sessions. As noted above, participant autonomy is prioritized throughout. See Multimedia Appendix 2 for an overview of the PA intervention. Of note, caregivers are required to be present for each PA session, either at home or in the same room as the participant, depending on the participant’s age, functional characteristics, and preferences (see the “Safety” section). If caregivers wish, they may participate in the PA sessions after completing the Get Active Questionnaire to ensure readiness and safety for engaging in PA, although the primary focus remains on the participant [78].

All PA sessions are delivered by a trained exercise professional who has completed a comprehensive >30-hour training program comprising both educational (eg, cancer and treatment protocols) and practical components (eg, exercise prescription, delivery, behavior change support, motivational communication skills). This training included the Thrive Health Cancer and Exercise program [79], as well as pediatric cancer and exercise [80] and adolescent- and young adult–specific modules [81]. Exercise professionals also attended trial-specific training covering topics such as oncology treatment and side effects, modifying PA based on treatment-related side effects, supporting movement, and intervention processes, delivered by the trial team. Finally, they completed a scenario-based competency training session, shadowed an existing group-based PA program in the community (ie, Pediatric Cancer Patients and Survivors Engaging in Exercise for Recovery [73,74]), and practiced delivering PA via videoconference to children aged 5-14 years without a history of cancer [82].

### Safety

To ensure safe delivery of the intervention and trial, several precautions are taken. Specifically, participants are medically cleared by one of their health care providers (eg, oncologist, nurse, physiotherapist) to confirm they can safely participate in PA sessions and physical function assessments. Participants’ personal and medical information is collected as part of baseline (week 0) participant- and caregiver-reported outcomes and reviewed by the trial team, including assessors and exercise professionals. If additional information is required, the trial team consults the participants and their caregivers, the participants’ health care team, and an exercise professional with >20 years of experience delivering PA to this cohort to provide safe exercise alternatives and modifications. PA sessions are led by trained exercise professionals (see the “The Intervention” section for their training). The exercise professionals follow a detailed protocol designed to support safe delivery of PA and enable necessary modifications. During sessions, they regularly check in with participants to ask how they are feeling and whether they wish to continue exercising, and they quickly adapt exercises based on participants’ responses. Additionally, exercise professionals have participants’ full address, phone number, and emergency contact information readily available, and follow established protocols in the event of a medical emergency. Two members of the trial team (AW, CCV, or Julia T Daun) also review 10% of PA sessions for each participant—see the “Implementation (Throughout)” section—using a standardized checklist covering multiple categories, providing comments specifically on safety. During assessments of physical function, see the “Assessments of Physical Function (week 0, week 12, week 24, and week 52)” section, similar safety strategies are followed. Specifically, assessors have experience conducting videoconference-based assessments with young adults and adults living with and beyond cancer [53,83,84], and have received study-specific training and practice assessing children without a history of cancer. As noted above, caregivers are present during assessments, assessors have access to participants’ medical and personal information, and 2 assessors are present to support data collection and implement emergency protocols if required.

### Data Collection

#### Assessment Timeline and Data Collection

As described above, assessments occur at baseline (week 0), postintervention (week 12), and 6- (week 24) and 12-month (week 52) follow-ups. See Table 1 for an overview of outcome measures and time points. Following the RE-AIM framework, multiple types of information are collected.

**Table 1:** Outcome measures and time points.

Reach (throughout)	Effectiveness (weeks 0, 12, 24, and 52)	Adoption (throughout)	Implementation (throughout)	Maintenance (weeks 12, 24, and 52)
Referral rate (numbers and sources) Number of participants excluded and reasons for exclusion Participation rate (number of participants consenting to participate divided by the number of eligible individuals referred) Personal and medical information (ie, sex, gender, and other factors [eg, age, cancer diagnosis, sexual orientation, race/ethnicity, cultural background, and socioeconomic status]) of participating participants and caregivers (collected through caregiver-reported outcomes at baseline only) Proportion of participants from rural versus urban locations	Device-measured PA^a^ levels Participant and caregiver questionnaires (PA, quality of life, symptoms, cognitive outcomes, and resource use) Assessments of physical function (aerobic endurance, lower body flexibility, shoulder flexion range of motion, balance, and functional mobility) Interviews with participants and caregivers to explore the benefits of the PA intervention (at week 12 only) Resource use Direct and indirect costs (costs of intervention delivery and trial management)	Sources of referral Differences in referrals across referring sites	Trial retention Adherence to the PA intervention Percentage of missing data Interviews with participants and caregivers to explore their perceptions of feasibility (at week 12 only) Intervention delivery time and cost Trial delivery time and cost Expertise of exercise professionals PA session fidelity Adverse events (safety)	PA levels at 6- and 12-month follow-ups PA referral into community programs Access/use of maintenance offerings PA referral implementation

^a^PA: physical activity.

#### Reach (Throughout)

Reach is assessed using data on referral rate (numbers and sources), number of participants excluded and reasons for exclusion, participation rate (number of participants consenting divided by the number of eligible individuals referred), personal and medical information (ie, sex, gender, and other factors such as age, cancer diagnosis, sexual orientation, race/ethnicity, cultural background, and socioeconomic status) of participating participants and caregivers, and the proportion of participants from rural versus urban locations. Where possible, information is also gathered from nonparticipating individuals via the consent-to-contact form and emails to assess reasons for trial refusal and to inform understanding of barriers to participating in the PA intervention and trial.

#### Effectiveness

##### Device-Measured Physical Activity (Week 0, Week 12, Week 24, and Week 52)

Device-measured PA (ie, step count) is assessed by having participants wear a Fitbit Charge HR activity tracker and complete a tracking log for 7 days. Participants are mailed the Fitbit and sent account information (generated by trial staff) via email. They are instructed to wear the Fitbit for 7 consecutive days, for at least 10 hours per day. Along with the Fitbit, participants complete a tracking log to record wear times (eg, when they put on or removed the Fitbit, and any PA performed throughout the day). At the end of the 7-day period, participants sync their Fitbit with their account, and caregivers scan or photograph the tracking log and send a copy to trial staff via email. Participants may wear the device between assessment periods if they wish, but these data are neither collected nor analyzed. Fitbits are returned once participants complete all assessments. The participant’s step count from the 7-day period is retrieved from their Fitbit account by the trial team and averaged for each time point. Fitbits have been shown to be a reliable and valid method for collecting device-measured PA data in pediatric populations with and without chronic conditions [85-88] and have been used successfully in pediatric participants with cancer [69,89].

##### Participant and Caregiver Questionnaires (Week 0, Week 12, Week 24, and Week 52)

*Personal and medical information* is collected at baseline (week 0) from both participants and caregivers to describe their characteristics. This includes participants’ biological sex, gender, race, ethnicity, location, age, diagnosis, and year of diagnosis, as well as caregivers’ highest level of education, employment status, and annual household income. The questionnaire was developed by the trial team.

*Self-reported PA* at weeks 0, 12, 24, and 52 is assessed using a modified version of the Godin-Shephard Leisure-Time Exercise Questionnaire (m-GLTEQ) to capture frequency and duration of aerobic, resistance, flexibility, and balance training [90]. Participants complete the m-GLTEQ for themselves, regardless of age, and caregivers complete the m-GLTEQ based on both their own PA and their child’s PA behavior. Specifically, participants and caregivers report the number of times per week they (and, in the case of caregivers, both themselves and their child) engage in mild, moderate, and strenuous PA during leisure time for a minimum of 10 minutes. At baseline, they also report PA levels before their (or their child’s) cancer diagnosis, and at all other time points, they report PA over the past month. The m-GLTEQ is scored by calculating the total amount of PA (time × intensity) for each intensity and type of activity and summing the totals. Intensity is weighted using metabolic equivalents of 3, 5, and 9 for light, moderate, and strenuous PA, respectively. Higher scores indicate greater PA behavior. Reports will include total moderate-to-vigorous PA as well as total PA. The m-GLTEQ has demonstrated high reliability and validity in pediatric populations with cancer [91,92].

*Quality of life* at weeks 0, 12, 24, and 52 is assessed by both participants and caregivers using the Pediatric Quality of Life Inventory (PedsQL) 4.0 Generic Core Scales and PedsQL 3.0 Cancer Module [93,94]. Participants aged 5-7, 8-12, and 13-18 complete the Young Child Report, Child Report, and Teen Report versions of the PedsQL 4.0 Generic Core Scales and PedsQL 3.0 Cancer Module, respectively. Caregivers also complete a proxy report evaluating their child’s quality of life using the corresponding PedsQL 4.0 Generic Core Scales and PedsQL 3.0 Cancer Module. Caregivers of participants aged 5-7, 8-12, 13-18, and 18+ complete the Caregiver Report for Young Children, Caregiver Report for Children, Caregiver Report for Teens, and Caregiver Report for Young Adults, respectively. The PedsQL 4.0 Generic Core Scale includes 23 questions across 4 domains of functioning: physical, emotional, social, and school. Items are scored on a 3-point scale for the Younger Child version (5-7 years): 0 (not at all), 2 (sometimes), and 4 (a lot), and on a 5-point scale for the Child (8-12 years) and Teen (13-18 years) versions, ranging from 0 (never) to 4 (almost always). After reverse scoring, items are linearly transformed to a 0-100 scale (0=100, 1=75, 2=50, 3=25, 4=0) and averaged to generate domain and total scores. Higher scores indicate a better quality of life. The PedsQL 3.0 Cancer Module participant and caregiver reports for young children (5-7 years) include 26 items across 8 domains: pain and hurt, nausea, procedural anxiety, treatment anxiety, worry, cognitive problems, perceived physical appearance, and communication. Items are scored on a 3-point scale: 0 (not at all), 2 (sometimes), and 4 (a lot). For participants and caregivers of children aged 8-12 and teens aged 13-18, the module includes 27 items across the same 8 domains, scored on a 5-point scale ranging from 0 (never) to 4 (almost always). For both versions, items are reverse-scored, linearly transformed to a 0-100 scale, and averaged, with higher scores indicating better quality of life and fewer problems or symptoms. Both the PedsQL 4.0 Generic Core Scale and PedsQL 3.0 Cancer Module have been previously used to evaluate quality of life among pediatric participants with cancer in PA interventions [51,94], and their scores have demonstrated high reliability and validity in this population17.

*Symptoms* at weeks 0, 12, 24, and 52 are assessed by both participants and caregivers using the Symptom Screening in Pediatrics Tool (SSPedi) and mini-SSPedi, which were developed for children receiving cancer treatment and undergoing hematopoietic stem cell transplant [95]. These tools capture symptoms that are currently bothering the participant, or that bothered them in the past day. The mini-SSPedi is used for participants aged 5-7 years [95], and the SSPedi is used for participants aged 8-18 years. The mini-SSPedi includes 15 items scored on a 3-point scale ranging from 0 (not bothered at all) to 2 (extremely bothered). The SSPedi includes 15 items scored on a 5-point scale ranging from 0 (not bothered at all) to 4 (extremely bothered). For both versions, item scores are summed, yielding a total score ranging from 0 (no symptoms) to 60 (worst possible), with higher scores indicating greater symptom impact. In both versions, participants and caregivers are also asked a final (16th) open-ended question to report any other issues that have recently bothered them (or, in the case of caregivers, their child). Additionally, caregivers of participants aged 5-18 years complete a proxy report SSPedi [95], which includes the same 15 items scored on a 5-point scale from 0 (not bothered at all) to 4 (extremely bothered). Similar to the participant versions, the caregiver proxy report SSPedi sums the 15 items to yield a total score ranging from 0 (no symptoms) to 60 (worst possible), with higher scores indicating greater symptom impact. The SSPedi has demonstrated reliability and validity for assessing symptoms among pediatric participants undergoing cancer treatment or hematopoietic stem cell transplant [95].

*Cognitive executive functioning* at weeks 0, 12, 24, and 52 is assessed by caregivers using the Behavior Rating Inventory of Executive Function, Second Edition (BRIEF-2) [96]. The parent proxy report form is used for all children and adolescents [97]. The BRIEF-2 includes 9 items covering major areas of executive function—behavior, emotion, and cognitive regulation—scored on a 3-point scale: 1 (never), 2 (sometimes), and 3 (often). Caregivers indicate how often their child has experienced each problem behavior over the past 6 months. Raw scores are calculated and summed to generate a score for each subscale. Additional scoring is applied for the inconsistency, negativity, and infrequency scales. The t scores and percentiles are also calculated for each raw scale score, and 90% CIs are computed to estimate the measurement error associated with the clinical scale, index, or t scores. Higher scores indicate greater cognitive impairment. The BRIEF-2 has been used with pediatric participants with cancer [98] and has demonstrated reliability and validity in this population 94.

*Resource use* at weeks 0, 12, 24, and 52 is assessed by caregivers using a modified version of the Pathways in Autism Spectrum Disorder, Phase III Resource Use Questionnaire for Adolescents (RUQ-A; November 30, 2016) [99]. Items have been adapted to the context of this trial and population. The modified RUQ-A assesses 7 types of resources that participants may use: (1) school; (2) mental health and psychological interventions; (3) rehabilitative services (eg, occupational therapy, physiotherapy); (4) child-focused recreational activities; (5) medications; (6) additional services; and (7) caregiver time associated with treatment and care. Caregivers are asked to indicate the frequency of use and costs associated with each resource their child accessed over the past 3 months. A final open-ended question allows caregivers to report any additional resources and associated costs not captured in the listed categories. The RUQ-A is not scored, and information on validity, interrater and test-retest reliability, and responsiveness is not summarized or statistically reported [99]. The RUQ-A is flexible, allowing questions to be adapted for the population of interest [99]. Although the RUQ-A has previously been used with caregivers of children with autism [99], to our knowledge, this is the first adaptation of the modified RUQ-A for pediatric participants with cancer.

##### Assessments of Physical Function (Week 0, Week 12, Week 24, and Week 52)

Each physical assessment is conducted by 2 trained exercise professionals via videoconference at the participant’s convenience, with a caregiver present. The assessments were selected based on their prior use with pediatric patients with cancer in person and for pragmatic reasons related to the feasibility of videoconference delivery [100].

*Aerobic endurance* at weeks 0, 12, 24, and 52 is assessed using the 2-minute step test [101]. Participants position themselves perpendicular to the camera, with their right leg facing the camera, and march in place for 2 minutes. They are instructed to lift their knees to hip height, so that their thighs are parallel to the floor. Assessors count the number of steps completed with the right leg (ie, the leg facing the camera). Participants also report their perceived exertion on a scale from 0 (nothing at all) to 10 (very, very hard; maximal; extremely strenuous exercise) [101]. A greater number of steps indicates higher aerobic endurance. Although this test has not previously been used with pediatric participants with cancer, it has been validated in young adults and adults living with and beyond cancer [52,53,83] and was deemed suitable due to the ease of videoconference administration.

*Lower body flexibility* at weeks 0, 12, 24, and 52 is assessed using the sit-and-reach test [102]. Participants begin by sitting on the edge of their chair and stretching each leg twice for 20 seconds. Next, they position the chair perpendicular to the camera, sit on the edge, and extend 1 leg at a time, keeping the ankle bent at 90°. Participants then hold a measuring device (eg, ruler or measuring tape) with both hands and lean forward as far as possible, reaching toward the toes of the extended leg while keeping the leg straight. Participants hold the maximum flexion position for 3 seconds, then measure the distance from the measuring device to the tip of their toes, recording the measurement to the nearest 0.5 cm. The test is performed twice on each leg, and the best score for each leg is recorded. Higher scores indicate greater lower-body flexibility. The sit-and-reach test has previously been used to assess flexibility among pediatric participants with cancer in person 108,109.

*Shoulder flexion range of motion* at weeks 0, 12, 24, and 52 is assessed using the shoulder flexion test [103,104]. Participants position their chair perpendicular to the camera, sit upright with feet flat on the floor, and face forward so that the shoulder being assessed is perpendicular to the camera. The camera is adjusted to capture the area from the participant’s belly button up to the height their hand would reach when extended overhead. Participants begin with their arms at their sides, palms facing inward (toward the hips), and thumbs pointing upward (toward the ceiling). While keeping the arm straight, participants raise it in forward flexion within the sagittal plane, avoiding compensatory movements (eg, arching the back or moving the arm away from the ear). Once the participant reaches their full range of motion, they hold the position while the assessor takes a screenshot. This procedure is repeated twice for each arm, and the average of both trials is calculated. After the assessment, the assessor measures the range of motion in degrees using a goniometer and anatomical landmarks (head of the humerus, midline of the humerus, and mid-axillary line). Higher scores indicate a greater range of motion. Although this test has not previously been used with pediatric participants with cancer, it has been validated in young adults and adults living with and beyond cancer [52,53,83] and was deemed suitable for videoconference administration.

*Balance* at weeks 0, 12, 24, and 52 is assessed using the flamingo balance test [105]. Participants stand barefoot with their arms crossed over their chest, placing each hand on the opposite shoulder. When ready, the assessor instructs the participant to stand on a leg of their choice while lifting the opposite leg, bending the knee so that the foot is behind them and not touching the standing leg. Timing begins when the foot is lifted and continues for a maximum of 45 seconds or until the participant loses balance. If balance is lost within the first 3 seconds, a second trial may be completed, with the better time recorded. The test is performed once on each leg unless a second trial is required. Longer times indicate better balance. The flamingo balance test has previously been used to assess balance among pediatric participants with cancer in person [106].

*Functional mobility* at weeks 0, 12, 24, and 52 is assessed using the 30-second sit-to-stand test [105] and the timed up and go test [107]. For the 30-second sit-to-stand test, participants sit on a chair with their arms crossed over their chest, hands on opposite shoulders. From this position, participants rise to a full standing position (knees straight) and return to a seated position, ensuring full contact with the seat before standing again. When the assessor says “ready-set-go,” participants complete as many sit-to-stand repetitions as possible within 30 seconds, and the total number is recorded. This test is completed only once. Higher scores indicate greater functional mobility. The 30-second sit-to-stand test has previously been used to assess functional mobility among pediatric participants with cancer in person [69,108-110]. The timed up and go test [107] is also used in this trial to assess functional mobility. Participants, with the help of their caregivers, set up a chair and measure 3 m (10 feet) from the chair, marking the distance with an object or piece of tape. Participants sit with their backs against the chair. On the “ready-set-go” cue, they stand up without assistance, walk as quickly as possible (without running) past the 3-m mark, turn around, and return to the chair. The assessor stops the timer once the participant’s back touches the back of the chair. Participants complete 1 practice trial, followed by 2 counted trials, the results of which are averaged. Lower times indicate greater functional mobility. The timed up and go test has previously been used to assess functional mobility among pediatric participants with cancer in person [106,111,112].

##### Interviews (Week 12)

Participants and their caregivers are invited to complete semistructured interviews. Interviews are conducted either one-on-one or as a dyad, depending on the participant’s and caregiver’s preference, by trained members of the trial team who are not involved in delivering the intervention. A semistructured interview guide is followed, in which the interviewer asks a series of open-ended questions with follow-up probes to elicit detailed responses. The interviews aim to gain a deeper understanding of participants’ and caregivers’ experiences with the PA intervention, including what they liked and disliked, its acceptability (satisfaction), feasibility, barriers and facilitators to participation, and plans for continued or future engagement in PA. The Capability, Opportunity, Motivation, and Behavior (COM-B) framework informed selected questions in the interview guide [113]. In addition, questions explore participants’ and caregivers’ desire for ongoing access to the PA intervention and other PA opportunities—see the “Maintenance (Week 12, Week 24, and Week 52)” section. Multimedia Appendix 3 presents the semistructured interview guide. All interviews are audio-recorded using a Sony ICD-PX240 or Sony ICD-PX470 recorder and transcribed verbatim.

#### Adoption (Throughout)

Adoption is assessed by tracking referral sources. Differences in referral rates are monitored across channels (eg, referral vs self-referral) and between the 2 sites, Alberta Children’s Hospital and Stollery Children’s Hospital.

#### Implementation (Throughout)

Implementation is being evaluated using data on indices of feasibility, including intervention delivery time, trial delivery time, required expertise, cost, fidelity of intervention delivery, and adverse events. Feasibility is defined by participant retention in the trial (ie, completion of assessments), adherence to the PA intervention (ie, percentage of PA sessions attended out of those offered), overall percentage of missing data, and participants’ and caregivers’ perceptions of feasibility, which are collected via qualitative interviews. For intervention delivery, the time required for delivering the PA intervention, conducting PA assessments (including administrative tasks), health care provider referral time, and the costs associated with implementation (eg, delivery and administrative support costs) are being tracked. Fidelity refers to the extent to which the intervention is delivered as intended and is assessed by randomly selecting 10% of PA sessions for each participant. These sessions are recorded and reviewed by 2 trial team members (AW, CCV, or Julia T Daun) who are not involved in delivering the PA or conducting assessments. Trial team members review the recorded sessions using an established checklist covering various categories, including safety, exercise tailoring and modifications, and adherence to the protocol. This process provides a percentage of sessions delivered as intended—meaning the PA program is followed with appropriate modifications, behavior change components are embedded, and delivery remains autonomy-supportive and tailored to each participant’s preferences. Fidelity checks also offer an opportunity to provide feedback to individual instructors if improvements are needed. Adverse events are tracked using a standardized reporting form capturing details such as date, timing, site/location, duration, severity, action taken, and outcome, following the Common Terminology for Adverse Events (CTCAE) version 5.0 [114]. Participants, caregivers, and exercise professionals may report adverse events; however, the responsibility for documenting each adverse event lies with the exercise professional and trial staff.

#### Maintenance (Week 12, Week 24, and Week 52)

Maintenance is assessed via semistructured interviews—see the “Interviews (Week 12)” section—to explore participants’ and caregivers’ desire for ongoing access to the PA intervention or plans (or both) for continued engagement in PA. In addition, participant- and caregiver-reported PA levels will be collected at follow-ups through the m-GLTEQ via REDCap, enabling exploration of sustained PA levels.

### Quality Improvement Cycles

Quality improvement cycles are one way to explore and refine implementation strategies so that, by the end of the trial, there is an optimized approach (including all required resources; [115]). Within this trial, the Consolidated Framework for Implementation Research has been fully operationalized and is used to categorize the data collected [116]. Quality improvement cycles are conducted every 6 months, during which the trial team reviews participants’ and caregivers’ personal and medical information, as well as intervention fidelity data, to ascertain the necessity of potential additional recruitment strategies to further diversify the sample. Additionally, a purposefully recruited subset of health care providers and exercise professionals who have been involved with the intervention and trial are selected to participate in 1:1 semistructured interviews, based on their role in referring to or delivering the PA intervention, respectively. These interviews are not conducted during every quality improvement cycle but are scheduled based on conversations among the trial team. Additional individuals are recruited during quality improvement cycles when there have been new health care providers who have referred, and exercise professionals involved in PA session delivery or conducting assessments of physical function (or in both scenarios). Interviews are conducted by trained members of the trial team who are not involved in intervention delivery. A semistructured interview guide is followed. The interviewer asks a series of open-ended questions, with follow-up probes, to gain a better understanding of whether additional resources are required to facilitate recruitment or whether further training is needed to support the delivery of the intervention by the exercise professionals. The semistructured interview guide can be found in Multimedia Appendix 3. All interviews are audio-recorded using a Sony ICD-PX240 or Sony ICD-PX470 recorder and transcribed verbatim. Data from quality improvement cycles are analyzed using descriptive statistics, examining recruitment rates and participants’ personal and medical information (eg, sex, gender, and other factors), as well as content analysis for interviews. These data are being used to optimize implementation by exploring whether additional resources are required to support recruitment (eg, posters, brochures, emails), to support health care providers to refer, and whether further training is needed to support exercise professionals.

Any modifications identified during quality improvement cycles are carefully considered by the trial team and advisory board (see the “Participant and Caregiver Involvement” section) and are implemented only when deemed necessary to enhance recruitment, increase participant diversity, support health care provider recruitment, or improve training for trial staff and the exercise professionals (or in all of the aforesaid scenarios). No modifications are made to PA session delivery, assessments of physical function, or other aspects of the intervention or trial that could influence effectiveness data.

### Participant and Caregiver Involvement

Participants’ and caregivers’ perspectives are deemed critical within this work. As such, an advisory board was developed 1 year into the trial, in August 2023, after several participants had completed the intervention. Currently, the advisory board comprises 1 participant (of note, initially 2 past participants comprised the board; however, 1 passed away), 2 caregivers, 1 health care provider, 1 community partner, and 4 trial team members (including the 2 coprincipal investigators). The advisory board meets quarterly by videoconference to discuss data collected in the quality improvement cycles (when relevant), provide advice regarding potential challenges, gaps, and opportunities within the PA intervention and trial implementation, advise on current and future dissemination opportunities, and contribute to adaptation and scaling discussions. At trial cessation, advisory board members will have the opportunity to review and comment on all data and will work with the trial team to contextualize the findings. All members have also contributed to, reviewed, and approved this manuscript.

### Data Analysis

Data will be analyzed and disseminated through conference presentations and manuscripts at multiple time points, including interim and trial cessation. To date, interim analyses have been conducted covering implementation metrics within the first 30 months, descriptive information, and individual changes in selected secondary outcomes pre- to postintervention [117]. No predetermined stopping rules were set within the context of the interim analyses.

At trial cessation, the full dataset will be analyzed. Single proportion inference tests and CIs will be used to determine the proportion of eligible participants who provided informed consent (or assent where appropriate) and adherence rates to the intervention components. Initial plans to analyze effectiveness data included generalized linear mixed models to examine changes over time in participants’ device-measured PA (primary effectiveness outcome) and in secondary effectiveness outcomes, multilevel modeling to examine site differences in relation to the primary and secondary outcomes, and inclusion and stratification of covariates (sex, gender, and other factors) to explore changes between cancer and blood disorder diagnoses and across age ranges (if sufficient power). Given the information presented in the “Sample Size” section, this trial is well below the sample size target. Thus, at trial cessation, alternative data analysis approaches will be considered. It is anticipated that the analysis will emphasize individual (idiographic) patterns and trajectories rather than relying solely on the more aggregated approach described above. Implementation data related to reach (referral rate, participation rate, participant personal and medical information, caregiver personal information, proportion of participants from rural vs urban locations), adoption (sources of referrals, differences in referrals across referring sites), implementation (trial retention, adherence to the PA intervention, percentage of missing data, intervention delivery time, expertise, PA session fidelity, trial delivery time, adverse events), and maintenance (participants’ and caregivers’ desire for ongoing access to the PA, participant- and caregiver-reported PA levels at follow-up time points) will be assessed using descriptive statistics via SPSS (IBM Corp) or R (R Foundation). The CONSORT diagram ([60]; Figure 1) will be completed to illustrate participant recruitment and retention in the trial.

All interviews will be transcribed verbatim, and transcripts will be managed and analyzed in NVivo (Lumivero) using conventional content analysis [118]. Each transcript will be read several times so the authors conducting the analysis can familiarize themselves with the data. Next, the transcripts will be coded. Labels (ie, codes) will be created to reflect key ideas. Codes will then be organized into groups and higher-order categories [118]. Interviews with participants and caregivers will be analyzed to explore participants’ and caregivers’ experiences within the intervention; acceptability, barriers, and facilitators to participation; feasibility; and their desire for ongoing access to the PA intervention, plans for continued engagement in PA; or all of these.

With respect to integrating quantitative and qualitative data, the initial plan included an embedded mixed-methods approach. Quantitative data will be reviewed, and qualitative data will be layered over to expand and provide further context to the situation, thereby complementing the findings [119]. This integration is an important component of mixed-methods research and will provide a more comprehensive understanding of the effects of the intervention, enhancing the depth and context of the findings [120]. Should the target sample size not be met, as noted above, the embedded mixed-methods approach may shift to feature the qualitative data; this will be determined via consultation with the trial team.

Additional analyses of selected trial data are planned and will cover health care providers’ experiences referring and not referring to the IMPACT intervention and trial, as well as exercise professionals’ experiences delivering the 1:1 PA sessions, conducting assessments of physical function, or both. These data will be analyzed using the fully operationalized Consolidated Framework for Implementation Research and conventional content analysis. These analyses will be conducted and published separately from the main trial results. Finally, given the amount of data collected within this trial, additional exploratory analyses may be conducted; these analyses will seek to answer additional research questions and will occur only after the interim and main analyses described above, based on discussion and agreement among the trial team.

## Results

This trial received funding in April 2022, and funding extensions were sought (due to slower and lower than anticipated recruitment) until March 2026. Participant recruitment commenced in March 2022 and will conclude in December 2025. Interim analyses were conducted in February 2025 and submitted in November 2025 [117]. As of October 2025, 105 patients have been referred (health care provider referral; n=90, 85.7%) or self-referred (n=15, 14.2%). Of the 105 referred participants, 42 (40%) scheduled a phone call with the trial team to receive more information, of which 26 (62%) provided informed consent; 16 enrolled in and completed the intervention, and 1 is currently actively in the intervention.

## Discussion

### Summary

PA is feasible, safe, beneficial, and recommended for pediatric patients with cancer [28,31]. Indeed, PA can improve physical, psychological, social, and cognitive outcomes, as well as overall quality of life [25-27,91,121-123]. Yet, pediatric patients with cancer face many barriers that make engaging in PA during treatment challenging. As a result, pediatric patients with cancer may not be active enough during treatment to accrue benefits [35,36]. To date, most PA interventions for pediatric patients with cancer have required participants to attend in-person PA sessions [31], which may be challenging due to treatment-related side effects, periods of isolation, variable treatment timelines, lack of PA opportunities, and geographical location [38-42]. PA delivered by videoconference may help overcome common barriers and be one way to offer PA to a small and geographically dispersed population [43]. However, only a few PA interventions have been delivered by videoconference for this population [45,46], and the effectiveness and factors influencing implementation for this population remain relatively unknown.

### Strengths

This manuscript details the protocol for our trial evaluating a PA intervention delivered by videoconference to pediatric patients with cancer (and pediatric patients with blood disorders) treated at the children’s hospitals in Alberta. Notable strengths of the PA intervention and trial include the following: first, the development process, which utilized an evidence-based [31,33] and patient-oriented approach [72], wherein extant data and resources were integrated with perspectives gathered through semistructured interviews with international experts and local health care providers; and second, the pragmatic nature of the intervention and trial, which could support a more rapid translation of findings to practice. Third, the broad eligibility criteria for participants ensure that all have access to PA, a recommended health behavior [124] and a basic right for all children and adolescents [125], and could help advance the field by including data from the range of cancers diagnosed. Fourth, by including patients with blood disorders, it is possible that evidence will be generated for the effectiveness of PA for pediatric patients with blood disorders, which is limited to date [68]. Relatedly, by offering PA to both pediatric patients with cancer and those with blood disorders—who are often treated on the same ward—this intervention and trial may support a shift toward a culture in which movement is supported and more readily available during treatment. Fifth, recruitment from 2 sites may overcome the often-reported limitation in this field of small sample sizes [50], enhancing the statistical power to further understand the relationship between PA and domains of functioning while exploring covariates (sex, gender, and other factors). Finally, the mixed-methods approach will provide a more comprehensive and nuanced understanding of both effectiveness and implementation by incorporating more than 1 perspective (ie, input from participants and caregivers) [126].

### Limitations

Notwithstanding the strengths of this work, there are some anticipated challenges. First, challenges in collecting device-measured PA data from participants during the 7-day Fitbit wear are foreseen (eg, forgetting to wear the Fitbit, forgetting to complete the tracking log). Although steps to minimize this have been taken (ie, additional reminders), it is plausible that the device-measured PA data may have high levels of missing data. Second, the volume of assessments may be burdensome to participants and caregivers. The decision was made to collect data for outcomes at various time points to help generate evidence for PA delivered by videoconference and its potential lasting impacts (ie, maintenance) in a field lacking research to date. Third, recruiting participants from multiple sites is an advantage, but it may increase the amount of time required for coordination and overall trial costs. Strong communication is required to ensure ongoing referral and engagement with health care providers at both children’s hospitals in Alberta. Fourth, although the heterogeneous nature of the population is a distinct advantage in terms of representing the breadth of cancer (and blood disorders) diagnoses, the variability could make it challenging to detect changes when analyzing data based on different disease trajectories, treatments, and treatment-related side effects. Relatedly, the single-arm design and varied treatment timelines of participants mean that some may receive the PA intervention during a time when they are on treatment and experiencing high symptom burden, and therefore, results may not be reflective of PA engagement alone. Without a control or comparison group, interpretation of findings will be cautious. However, alternative data analysis strategies may be beneficial to truly understand the impact of IMPACT, such as collecting, analyzing, and integrating both the quantitative and qualitative data to provide context for findings. Finally, although individualized and tailored PA sessions are important to ensure safety, support participants’ autonomy, and enhance the likelihood of behavior change, challenges in succinctly reporting what was done in each session are anticipated. While exercise professionals are keeping detailed tracking logs for each participant on the components delivered, behavior change techniques used, participants’ fatigue and energy before/during/after PA sessions, and other notes (eg, side effects reported and what the participant liked/did not like), it may be difficult to distill and report the details of the PA sessions in a way that ensures they can be replicated by others. The decision to offer a tailored intervention ensures personalized care and the potential to optimize benefits for each participant.

### Conclusions

Although PA is feasible, safe, beneficial, and recommended for this cohort, little is known about the effectiveness and implementation of PA via videoconference (see [45,46] for notable exceptions). Results from this trial may provide real-world data and insight into factors influencing the implementation of a PA intervention delivered by videoconference for pediatric patients with cancer and blood disorders. In particular, findings may support the use of videoconferencing to deliver PA to support pediatric patients to move more and experience the benefits of PA during treatment.

## Data Availability

The datasets generated or analyzed from the trial described may be available, upon reasonable request (AW), from the corresponding author at trial cessation.

## Appendix

Multimedia Appendix 1 Reporting checklists.

Multimedia Appendix 2 Physical activity intervention program.

Multimedia Appendix 3 Interview guide.

## Abbreviations

BRIEF-2: Behavior Rating Inventory of Executive Function, Second Edition
COM-B: Capability, Opportunity, Motivation, and Behavior
CONSORT: Consolidated Standards of Reporting Trials
CTCAE: Common Terminology for Adverse Events
GRAMMS: Good Reporting of a Mixed Methods Study
HREBA: Health Research Ethics Board of Alberta
IMPACT: IMplementation of Physical Activity for Children and adolescents on Treatment
m-GLTEQ: modified version of the Godin-Shephard Leisure-Time Exercise Questionnaire
PA: physical activity
PedsQL: Pediatric Quality of Life Inventory
RE-AIM: reach, effectiveness, adoption, implementation, maintenance
REDCap: Research Electronic Capture
RUQ-A: Resource Use Questionnaire for Adolescents
SPIRIT: Standard Protocol Items: Recommendations for Interventional Trials
SSPedi: Symptom Screening in Pediatrics Tool
StaRI: Standard for Reporting Implementation Studies
TIDieR: Template for Intervention Description and Replication
