# Supplementary material for: IMplementation of Physical Activity for Children and adolescents on Treatment (IMPACT) for Cancer Diagnoses in Alberta: Protocol for a Single-Arm, Mixed-Methods, Hybrid Effectiveness-Implementation Trial
Source: JMIR Res Protoc. 2025 Dec 17;14:e59302. doi: 10.2196/59302 (PMC12756665; doi:10.2196/59302)
Supplement: Multimedia Appendix 2 [file resprot_v14i1e59302_app2.pdf]

Supplementary File 2. Overview of the PA intervention

IMPACT

IMplementation of Physical Activity for Children and adolescents on Treatment

Online Physical Activity Guide

[FOR PA SPECIALISTS TO FOLLOW]

Postural Cues

Cueing Shoulder Retractions

1) Have participants stand normally with their back against the wall. They may need to step their feet away from the wall to keep a flat back.
2) Have participants rotate their palms so thumbs point out and palms face forwards. They should feel this automatically improve posture - chest lifts, shoulders back and down. Shoulder blades may become flat with the wall. Ensure all participants can feel this.
3) Participants should extend their arms out in front of them. This should cause the shoulder blades to no longer sit flat against the wall. Have participants pull their arms back slightly so the shoulders move back and down. Shoulder blades should become flat with the wall.
4) Once participants feel this, have them repeat a few more times. Participants should hold the retractions for 3-5 seconds before releasing.

Cueing Core Engagement

1) Participants should lie on the ground with their feet planted on the floor. Have them place their fingers on their abdomen, below their belly button, just inside their pelvis. ** can also be completed with their back against the wall if they have difficulty getting down to the floor
2) Cue participants to cough a couple times. They should feel their abdominal muscles under their fingers contract. These are the muscles they want to engage during exercises in class.
3) Participants should place their pinkies on their pelvis (bony part on the front of their hip) and their thumbs on their low ribs. Cue participants to think about engaging their core and pulling these two landmarks together. They should also feel their lower back pressing into the floor or wall.
4) Once participants can feel their abdominal muscles engaging. Have them repeat this a few more times, holding the engagement for 3-5s each time.

Warm-Up

Choice to complete: standing, seated, in bed, or on the ground.

Cardio

- Walking/jogging on spot
- High knees
- Bum kicks
- Speed skaters
- Lateral jumps
- Jumping jacks
- Seated cardio

Upper body

- Shoulder rolls
  - Forwards/backwards
- Arm circles
  - Forwards/backwards
- Is, Ys, Ts
- Wall angels
- Chest openers (hug arms together and open arms wide, squeezing shoulder blades together)
  - Switch top/bottom arm each time
- Wrist rotations
- Side reach (arm up and reach over to opposite side)

Lower body

- Hip hinges
- Hamstring/calf stretch
- Squats
- Side lunge
- Glute clenches
- Hip openers (open the gate)

Cool-Down/Stretching

Choice to complete: standing, seated, in bed, or on the ground.

Lower body

- Knees to chest (rotate ankle)
- Hamstring/calf stretch
- Figure 4
- Windshield washers (feet hip distance apart, feet on ground, rotating knees from one side to another)
- Quad stretch

Upper body

- Cross-body shoulder stretch
- Overhead triceps stretch
- Chest stretch
    - Option I: Grasp hands behind back and roll shoulders down
    - Option II: Chest openers (hug arms together and open arms wide, squeezing shoulder blades together)
        - Switch top/bottom arm each time
- Wrist stretch (flexion and extension)
- Trapezius stretch
    - Arms/hands come out to ‘W’ and then close together like holding onto a beach ball
        - Progression I: drop chin to chest
        - Progression II: with chin dropped, rotate head from side to side

Exercise Program Quick Overview
[DESIGNED FOR CHILDREN]

Week 1 & 2 Circuit Overview: Animal Theme *introduce education topic 1*

**Week 1 (45 Sec x 2 sets)**	**Week 2 (45 Sec x 2 sets)**
Circuit 1:	Circuit 1:
a) Lower Body	a) Lower Body
b) Upper Body	b) Balance
c) Cardio	c) Cardio
Circuit 2:	Circuit 2:
a) Postural Cues/Upper Body	a) Postural Cues/Upper Body
b) Balance	b) Upper Body
c) Cardio	c) Cardio
Circuit 3 (on mat):	Circuit 3 (on mat):
a) Core Engagement	a) Glutes
b) Glutes	b) Core
c) Glutes	c) Back Core

Week 3 & 4 Circuit Overview: Animal Theme *introduce education topic 2*

**Week 3 (45 Sec x 2-3 sets)**	**Week 4 (45 Sec x 2-3 sets)**
Circuit 1:	Circuit 1:
a) Lower Body	a) Lower Body
b) Postural Cues/Upper Body	b) Upper Body
c) Glutes	c) Lower Body/Balance Focus
d) Cardio	d) Cardio
Circuit 2:	Circuit 2:
a) Lower Body	a) Upper Body
b) Upper Body	b) Balance
c) Cardio	c) Cardio
Circuit 3 (on mat):	Circuit 3 (on mat):
a) Upper Body	a) Lower Body
b) Core	b) Side Core
c) Back Core	c) Core

Animal Theme Exercise Ideas (week 1-4):

Warm-up/cool down: Elephant trunk, mad cat, butterfly, cobra, elephant dangle (forward fold), giraffe stretch, pigeon lunge
Lower body: Frog walk, horizontal frog jump, vertical frog jump, gorilla walk, bunny hop, low duck walk, donkey kicks, bird-dog, elephant fold (deadlift w arms up)
Upper body: Alligator crawl, crab walk
Cardio: duck walk, dinosaur walk, lion sprint, mountain climbers
Core: Bear crawl, cocoon sit up, butterfly sit-up, Inchworm
Balance: silly monkey, drinking bird

Week 5 & 6 Circuit Overview: Superheroes theme *introduce education topic 3*

**Week 5 (1 min x 2 sets)**	**Week 6 (1 min x 2 sets)**

Circuit 1:

a) Lower Body
b) Upper Body
c) Balance
d) Cardio

Circuit 2:

a) Upper Body
b) Glutes
c) Cardio

Circuit 3 (on mat):

a) Glutes
b) Core
c) Side Core

Circuit 1:

a) Lower Body
b) Upper Body
c) Balance
d) Cardio

Circuit 2:

a) Lower Body
b) Upper Body
c) Cardio

Circuit 3 (on mat):

a) Core
b) Glutes
c) Glutes

Week 7 & 8 Circuit Overview: Superheroes Theme

Week 7 (1 min x 2-3 sets)

Circuit 1:

a) Lower Body
b) Upper Body
c) Balance
d) Cardio

Circuit 2:

a) Upper Body
b) Lower Body
c) Upper Body
d) Cardio

Circuit 3 (on mat):

a) Glutes
b) Core
c) Back Core

Week 8 (1 min x 2-3 sets)

Circuit 1:

a) Lower Body
b) Upper Body
c) Balance
d) Cardio

Circuit 2:

a) Upper Body
b) Lower Body
c) Glutes
d) Cardio

Circuit 3 (on mat):

a) Upper Body
b) Side Core
c) Core

<u>Superhero Theme Exercise Ideas (week 5-8):</u>

Warm-up/cool down: Superhero morning stretch, Warrior pose
Lower body: Hulk squats, Iron man lunge
Upper body: Supermans, Spiderman push-ups, Batman/Batwoman Is,Ys,Ts
Cardio: Super speed quick-feet, Wolverine walk, Moon walk
Core: Superhero crunch, Spiderman side plank
Balance: Captain America single leg pose

Week 9 & 10 Circuit Overview: Disney Movie Theme *introduce education topic 4*

Week 9 (1 min x 2-3 sets)

Circuit 1:

a) Lower Body
b) Upper Body

Week 10 (1 min x 2-3 sets)

Circuit 1:

a) Lower Body
b) Upper Body

c) Balance
d) Cardio

Circuit 2:
d) Lower Body
e) Upper Body
f) Core
g) Cardio

Circuit 3 (on mat):
a) Upper Body
b) Glutes
c) Core

c) Glutes
d) Cardio

Circuit 2:
d) Lower Body
e) Upper Body
f) Upper Body
g) Cardio

Circuit 3 (on mat):
a) Core
b) Back Core
c) Side Core

Week 11 & 12 Circuit Overview: Disney Movie Theme *introduce education topic 5*

Week 11 (1 min x 2-3 sets)

Circuit 1:
a) Lower Body
b) Upper Body
c) Glutes
d) Cardio

Circuit 2:
a) Upper Body
b) Lower Body
c) Balance
d) Cardio

Circuit 3 (on mat):
a) Upper Body
b) Lower Body
c) Core

Week 12 (1 min x 2-3 sets) *Option to use 'Create Your Own Adventure' Template

Circuit 1:
a) Lower Body
b) Upper Body
c) Cardio

Circuit 2:
a) Lower Body
b) Upper Body
c) Upper Body
d) Cardio

Circuit 3 (on mat):
a) Glutes
b) Core
c) Back Core

<u>Disney Theme Exercise Ideas (week 9-12):</u>

Warm-up/cool down: Mickey skip, Donald duck walk
Lower body: Aladdin sumo squat, Bambi reverse lunge
Upper body: Starwars Jedi lateral raise, Simba bicep curl
Core: Cinderella crunch, 101 Dalmatian core (bird dog)
Cardio: Prince/Princess waltz, Mickey skip

Exercise Program Quick Overview
[DESIGNED FOR ADOLESCENTS]

Week 1 & 2 Circuit Overview: *introduce education topic 1*

Week 1 (45 Sec x 2 sets)

Circuit 1:
- d) Squat to Chair
- e) Wall Push-ups
- f) Cardio: March or High Knees

Circuit 2:
- d) Scapula retraction (technique + practice) – progress to Seated "row" w/ bands day 2 if have them)
- e) Single Leg Stance balance
- f) Cardio: Butt Kicks

Circuit 3 (on mat):
- d) Core Engagement / Day 2: Pelvic Tilt
- e) Glute Bridge
- f) Day 2: Clamshells

Week 2 (45 Sec x 2 sets)

Circuit 1:
- d) Squat – ball wall squat if have or Wall Squat without ball
- e) Balance: Tandem Walk
- f) Cardio: Low/Jumping Jacks

Circuit 2:
- d) Wall Angels
- e) Wall Pushups
- f) Cardio: 2 feet in, 2 feet out

Circuit 3 (on mat):
- d) Glute Bridge – hold longer (5-10 sec hold)
- e) Deadbug with Heel drops
- f) Bird Dog

Week 3 & 4 Circuit Overview: *introduce education topic 2*

Week 3 (45 Sec x 2-3 sets)

Circuit 1:
- e) BW Squat OR (Ball) Wall Squats
- f) Lat pulldown (band or BW) OR Wall Angel
- g) Standing Hip Abduction
- h) Cardio: wall mountain climber OR High Knees

Circuit 2:
- d) Standing Hamstring Curls
- e) Seated Row w/ Band or Scapular Retractions
- f) Cardio: Speed Skaters

Circuit 3 (on mat):
- d) Chest press
- e) Deadbug with heel drops or small movements with opposing arms/legs
- f) Bird Dog

Week 4 (45 Sec x 2-3 sets)

Circuit 1:
- e) Reverse Lunge
- f) Single arm Bent Over Row
- g) Calf Raises (Balance focus)
- h) Cardio: Butt kicks w/ arm movement

Circuit 2:
- d) Push-ups (wall, edge of coffee table, ottoman, couch, chair)
- e) Balance: Single Leg Alphabet
- f) Cardio: "the Jess"

Circuit 3 (on mat):
- d) Hamstring Bridge with steps outs
- e) Side Plank with hip lift
- f) Leg Lowers

Week 5 & 6 Circuit Overview:

Week 5 (1 min x 2 sets)

Circuit 1:
- e) Reverse Lunge OR Split Squat

Week 6 (1 min x 2 sets)

Circuit 1:
- e) Squat (option to add weight)

f) Reverse Fly (BW to start w/ technique – progress to bands/DB if ok)
g) Balance: Tree Pose
h) Cardio: Lo/Hi Jumping Jacks OR Shadow Boxing

Circuit 2:

h) Chest Press with band or 'weights'
i) Monster Walk/Standing Hip Abduction
j) Cardio: Speed Skaters

Circuit 3 (on mat):

d) Glute Bridge
e) Dead Bugs (adding arms)
f) Side Plank Hip Lifts

f) Seated Row OR Standing Bent-over Row
g) Tandem Walk OR S/L Balance
h) Cardio: High Knee – cross-body crunch

Circuit 2:

h) Side Lunge
i) Lateral Raises (Bands, 'weights')
j) Cardio: 2 out/2 in (quick feet)

Circuit 3 (on mat):

d) Plank (wall, knees, toes)
e) Glute Kickbacks
f) Clamshells

Week 7 & 8 Circuit Overview: *introduce education topic 3*

Week 7 (1 min x 2-3 sets)

Circuit 1:

e) Sumo Squat
f) Push-ups – progression from previous
g) Balance: "Drinking Bird"/S/L Forward Bend
h) Cardio: Mountain Climbers

Circuit 2:

e) Reverse Fly
f) Calf Raises
g) Bicep curl; Triceps Kickback (30sec each)
h) Cardio: Choice!

Circuit 3 (on mat):

d) Glute Bridge March
e) Dead Bug – option to add ball OR pillow
f) Bird Dog

Week 8 (1 min x 2-3 sets)

Circuit 1:

e) Squat + Shoulder Press (OR weighted squat)
f) Lat-pulldown OR Wall Angel
g) Balance: S/L side kick (Abduction)
h) Cardio: Hi/Lo Jumping Jacks

Circuit 2:

e) One Arm Row + Triceps Kickback
f) Side Lunge
g) Standing Glute Kickback
h) Cardio: Choice!

Circuit 3 (on mat):

d) Chest Fly
e) Side Plank
f) Plank (wall, knees, or toes)

Week 9 & 10 Circuit Overview: *introduce education topic 4*

Week 9 (1 min x 2-3 sets)

Circuit 1:

e) Box Step-ups
f) 1-arm Band Lateral Raise
g) Balance: S/L Ball Pass OR Wobble Board/unstable surface
h) Cardio: Speed skaters

Circuit 2:

Week 10 (1 min x 2-3 sets)

Circuit 1:

e) Sumo Squat (option to add weight)
f) DB Chest Pullover
g) Standing hip abduction
h) Jumping Jacks OR Mountain Climber

Circuit 2:

a) Band Bent-over Row
b) Calf Raises
c) Band Pallof Press
k) Cardio: Quick Toe Taps

Circuit 3 (on mat):
d) Chest Press on Mat
e) Glute Bridge Kickouts
f) Dead Bugs (option to add stability ball)

a) Lunge – forward OR walking
b) Reverse Fly
c) Bicep-curl + Shoulder press
k) Cardio: High Knee – cross-body crunch

Circuit 3 (on mat):
d) Plank
e) Bird Dog
f) Side Crunch

Week 11 & 12 Circuit Overview: *introduce education topic 5*

Week 11 (1 min x 2-3 sets)

Circuit 1:
e) Step-Ups OR Squats
f) Shoulder Press OR Lateral Raises
g) Monster Walk
h) Cardio: 2ft in, 2ft out

Circuit 2:
e) Bent-over Row + Kickback
f) Side Lunge
g) Single-leg Forward Bend ('drinking bird'
h) Cardio: Choice!

Circuit 3 (on mat):
d) Chesty Fly
e) Hamstring Curls
f) V-sit

Week 12 (1 min x 2-3 sets) *Option to use 'Create Your Own Adventure' Template

Circuit 1:
d) Squat + Shoulder Press (OR weighted squat)
e) Band Lat-pulldown
f) Cardio: Speed skaters

Circuit 2:
e) 'Around the World' Lunges
f) Pushups
g) Bicep Curl – Triceps Kickback (30sec each)
h) Cardio: Choice

Circuit 3 (on mat):
d) Glute Bridge March OR S/L Glute Bridge
e) Plank (added challenge: Plank Up-Down)
f) Bird Dog – all 1 side

CREATE YOUR OWN ADVENTURE

For this session, you will create your own workout. Attached are lists of exercises that fall within the categories of upper body, lower body, core, and cardio. Pick an exercise that uses the major muscle groups for the first two circuits, then add in arms/accessory muscles once the big upper body muscles have been selected.

Circuit 1
Lower Body:
Upper Body - Back:
Balance
Cardio:

Circuit 2
Lower Body:
Upper Body - Chest:
Accessory Muscle: i.e. shoulders, triceps, biceps, calves
Cardio:

Circuit 3
Core: Hip/Glutes (eg. Bridges):
Core: Front:
Core: Back OR Oblique:

Exercises Broken Up by Muscle Group

Upper Body

Chest
1. Push Ups
2. Chest Press
3. Chest Fly

Back
1. Rows
2. Lat Pulldown
3. Reverse Fly

Arms
1. Bicep Curl
2. Triceps Extension or Kickback
3. Shoulder Press
4. Lateral Raise

Core

Front Core
1. Dead Bugs
2. Plank
3. V-sit

Back Core
1. Fire Hydrants
2. Birddogs

Oblique
1. Side Plank
2. Side Crunch
3. Side Plank – Hip lifts
3. Isometric T Hold/Pallof Press
4. Side Crunch Leg Lift

Lower Body

Quad
1. Squats or Sumo Squats
2. Lunges
3. Step Ups
4. Seated Leg Extensions

Hamstring
1. Hamstring Bridge Progression
2. Standing Hamstring Curls
3. Hamstring Curls on ball (Prone or Supine) w/ Ball
4. Seated Hamstring Curls

Glute
1. Glute Bridge
2. Donkey Kicks
3. Side Steps with Band (Glute Medius)
4. S/L Hip Abduction (standing side kick)

Cardio
1. 2 in 2 out
2. Skaters
3. High knees
4. Butt kicks
5. Jogging/marching
6. Jumping jacks
7. Mountain climbers
8. “the Jess” 1/2
9. Seated cario

Upper Body Exercises

Bent over Row	Hinge over at hips slightly, ensuring core is engaged and shoulder blades are set. Hold dumbbells with arms straight and squeeze the shoulder blades back and down, bringing hands back towards the mid-chest. Can be completed standing with band if there is concern about low back.
Bicep Curl	Rotate the hands while completing the bicep curls. Palms start facing thighs and end facing shoulders.

Bicep Curl and Press	Cue shoulder retraction and pelvic tilt. Make sure participants are not arching through the low back while completing this exercise.
Chest Fly	Keep a soft bend in the elbows and only open the arms until they are parallel with the floor.
Chest Press	Lie with back flat on the bench and core engaged. Keep the weights over the chest (not over the face).
Front Raise	With hands at side or slightly in front, raise hands up with palms down to shoulder height. Return to start.
I's, Y's, T's	Ensure the low back is pressed into the bench during this exercise. Participants should rotate between the I, the Y, and the T.
Lateral Raise	Keeping a pronated grip, open the arms to the side. Keep shoulders down and the neck relaxed
Lat Pulldown	Cue shoulder retraction and pelvic tilt. Make sure participants are not arching through the low back while completing this exercise.
Push Up	Easy: Wall<br>Medium: Incline (hands on bench)<br>Hard: On floor from knees or toes
Reverse Fly	Ensure core is engaged and shoulder blades are set before hinging over at waist slightly. Holding light weights, lift arms out to side and squeeze the shoulder blades together before repeating for the next repetition.
Row + kickback	This exercise can be completed single arm (on the bench), or with both arms. Ensure participants are keeping a flat back and shoulders stay level while completing the row.
Scapular retraction and shoulder alphabet	Rolling shoulder blades onto back, shoulders away from ears. Raise one arm and begin spelling the alphabet with an open palm, ensuring retraction the entire time (resting and resetting if form slips).
Seated Row	Sit on the bench and loop the resistance band around the feet. Keep the core engaged and shoulders back and down while pulling back on the band. Cue shoulder retractions from the previous session.
Shoulder Press	Keep the core engaged as the weights are lifted overhead. Cue the pelvic tilt practiced at the beginning of session.
Single arm row	Progress participants to a single arm row with the hand and knee on the bench. Ensure participants are keeping a flat back and shoulders stay level while completing the row.
Standing Row	Cue shoulder retractions from the beginning of sesssion. Loop the resistance band around bar (by mirror), standing staggered stance. Keep the core engaged and shoulders back and down while pulling back on the band.
Triceps Kickback	Easy: completed single arm like the SA row<br>Hard: completed with both arms at the same time, bent over
Triceps Press down	Place one end of a band on the wall and hold in place. Place free arm at a 90-degree angle while holding band, and straighten arm while ensuring shoulder joint does not move. Return to starting position.

Lower Body Exercises

Ball Squat	Place ball in the small of the back. Lean weight back into the ball and complete a squat. Only go as deep as feels comfortable on the joints.
Calf raises	Press weight through the ball of the foot. DO NOT want to roll out onto the baby toe edge of the foot. Standing beside the wall is an option for balance concerns.
Donkey Kicks	Ensure the low back is not sagging while participants extend the leg. These can be completed on the wall if the participant is unable to get down to the floor. Place weight in the bend of the knee if exercise needs to be more challenging.
Fire Hydrants	In tabletop position, ensure core is engaged and back is flat. Lift one knee off of the ground and open hip, ensuring that hips remain flat. Pause at top of movement before returning to start. Can be completed on the wall if difficulty getting to floor
Glute Bridge (with or without kick outs)	Hip lift off of the ground, pause for a second at top and release back down to mat. Ensure core is engaged throughout exercise. For kick outs: Lift one foot off ground, extend knee and return to ground before repeating with other leg.
Glute kickbacks	With arms extended and on toes, place the handle of a resistance band around one foot. Keep appropriate tension by holding the band in place to Kick straight back on one leg. Can also be done in tabletop position on ground.
Lunges	One foot in front of the other, alternating legs or walking
Prone Hamstring curl	Lying on stomach, place an exercise ball in between ankles. Squeeze ankles against ball and curl in towards body.
Seated Hamstring Curl	Sit on chair with both feet planted on the ground, and hands wherever comfortable. Keeping one foot planted, slowly curl one heel back behind, or up towards the bottom of the chair. Can add a band for increased difficulty.
Seated Leg Extension	Sit on chair with both feet planted on the ground, and hands wherever comfortable. Keeping one foot planted, slowly lift the other up off the ground, with toes pointing toward the ceiling, squeezing through the quad
Side Lunges	Step sideways, bending one knee into a lunge while pushing hips back and keeping heel on ground. Leave other leg straight
Side Steps with Band	Easy: do not cross bands<br>Medium/Hard: cross bands<br>Suggest participants take 10 steps in each direction, alternating throughout the set.
Split Squats	One leg in front of the other. Ensure front heel remains on the ground to prevent unnecessary tension through knee. Complete over by the wall if balance is a concern. If this bothers the knees, complete squats or leg extensions instead.
Standing Hamstring curl	Create a loop with the band, placing one foot inside. Provide tension on the band by stepping on it with free foot. Curl foot with band looped around towards glutes, relax and repeat

Step Ups	Complete with a tempo of 2 seconds up, 2 seconds down.<br>Participants can change the step height to match their comfort level
Sumo Squats	Wide stands with toes angled slightly outwards. Ensure participants are engaging the glutes to keep the knees from sagging inwards.
Supine Hamstring curl	Easy: Hips on Ground<br>Medium: Lift hips off the ground, rest them back on the ground between reps<br>Hard: Lift hips off the ground and keep hips up throughout the set

Core Exercises

Bird dog	Easy: one limb at a time<br>Medium: opposite arm and leg, toe stays on the ground
Dead bug	Easy: knees bent, one limb at a time<br>Medium: knees bent, opposite arm and leg at the same time<br>Cue pelvic tilt
Donkey Kicks	Ensure the low back is not sagging while participants extend the leg. These can be completed on the wall if the participant is unable to get down to the floor.
Glute Bridge	Hip lift off of the ground, pause for a second at top and release back down to mat. Ensure core is engaged throughout exercise.
Isometric T hold	Wrap a band around the bar and step out to an appropriate resistance level. Ensure hips and shoulders are square. Arms can be against chest or be extended directly in front of chest. Don't let participants over or under rotate to compensate against the resistance band.
Plank	Easy: on wall<br>Medium: on floor from knees<br>Hard: on floor from toes *the farther the feet are apart, the more stable the stance
Side Crunch Leg Lift	Easy: Lay on side body, head resting on ground or in hands, slowly lift top leg up and lean toward leg<br>Medium: Lift both legs up<br>Hard: Lift both legs up and hold
Side Plank	Easy: Wall<br>Medium: on floor from knees<br>Hard: on floor from staggered feet
V Sit	While seated, lean back until core is engaged. To make more challenging- lean back further or lift feet off ground. Make sure low back is not in any pain and chest is proud.
Clamshells	Lie on side with arm bent to use as pillow. Have knees stacked and bent to 90 degrees. While keeping feet together open up the top hip. Ensure that top hip does not roll back. Alternative: standing leg abduction if participant does not want to get down on floor.

Balance Exercises

Drinking bird	Lift one leg and opposite arm into straight line before hinging over at waist as far as comfortable and that allows the participant to return to standing without needing to place foot on ground. Can be completed by the wall for safety. Make sure the upper body and leg are moving in one straight line.
Single leg balance	Let participants move over toward the wall if balance is a concern.
Single leg balance with alphabet	Use free leg to spell alphabet with the foot
Single leg balance with eyes closed	
Tandem walking	Walk heel to toe in a line. If participants feel more comfortable, they can walk beside a wall
Tree Pose	Easy: toe on ground<br>Medium: foot on calf<br>Extra Challenge: look at the ceiling instead of the floor

[EDUCATIONAL TOPIC OVERVIEW: the topic examples to follow will be tailored to ensure the content is age appropriate. As well, additional resources will be added if/when required (ensuring appropriate research ethics approvals are obtained).]

SAMPLE

Education Topic One: Principles of Physical Activity

This content will be introduced verbally or by using a worksheet following the content provided below, which will be presented in an age-appropriate manner (e.g., use of coloring activities, journaling, etc.).

This education session is aimed at providing participants with information on the benefits of physical activity. This session will also discuss the different types of physical activity, the FITT principle, general exercise principles, the importance of a warm-up and cool-down, monitoring intensity, and safe exercise guidelines.

SAMPLE

There is evidence that physical activity is a safe and beneficial for young people affected by oncological and hematological diseases.

Benefits of being active can include:

Psychological and Emotional

- Reduced levels of anxiety, stress, and depression
- Improved self-esteem
- Improved body image
- Positive changes in feelings of control
- Opportunity to meet new friends/social support
- Increased energy and overall improvements in quality of life

Physical

- Reduced levels of fatigue
- Improved health and fitness
- Improved functional capacity, daily life becomes easier (i.e., climbing stairs)
- Improved immune system
- Helps maintain bone mineral density (i.e., helps maintain bone strength)
- Helps improve and maintain flexibility
- Lowers blood pressure (i.e., lowers the pressure of blood against blood vessels, which improves health)
- Promotes good posture and balance
- Achieve a healthy body weight and composition (build muscle, reduce fat)
- If you are on active treatment:
    - Reduce treatment-related pain, nausea
    - Diminishes impact of treatment-related symptoms

SAMPLE

What is the most important benefit of being active **for you**?

____

PHYSICAL ACTIVITY: THE FUNDAMENTALS

Physical Activity: Activity that is part of daily living. Examples include: playing at recess,

playing outside, going for a walk, and chores around the house.

Exercise: Planned and structured physical activity that is done to improve at least one aspect of physical fitness (strength, endurance, cardiovascular fitness (i.e., heart health) or flexibility).

There are different types of exercise that are associated with specific health-related benefits:

Aerobic Exercise

- The heart and lungs are responsible for the delivery of oxygen and nutrients needed to fuel working muscles. Aerobic training, also known as `cardio` training improves endurance, which is the ability to sustain repetitive muscular contractions over long durations (e.g., walking, biking, running).
- By doing more of this type of exercise, you will become less tired during and after your activities, exercises or sports.
- It is a key component in maintaining healthy heart and lung function

Resistance Exercise

- Resistance training (also known as strength training or weight lifting) is the method used to increase strength, and build strong, healthy muscles.
- There are many types of equipment that make resistance training possible. You can use weights, resistance bands, or other activities.
- Improvements in body composition are seen with resistance training as well as improvements in metabolism, with increase calorie burning muscle fibers.

SAMPLE

Flexibility/Stretching

- Flexibility is an important component of physical fitness that allows you to bend, twist, turn and stretch. Flexibility training (stretching) ensures you maintain a full range of motion in your joints.
- Stretching can be done daily (incorporated into your warm-up or cool-down) and each stretch should be held for about 20 seconds. When stretching, remember to breathe, do not bounce, and only stretch to the point of slight discomfort, never pain.

Exercise programs often follow the FITT Principle:

FREQUENCY – How often you exercise.

- Example: 3 times / week

INTENSITY – How hard you work during exercise.

- Example: 4/10 on the Rating of Perceived Exertion Scale

TIME – How long you exercise during each session. ***Can be cumulative!

- Example: 30 minutes or 3 x 10 minutes

TYPE – The mode of physical activity.

- Example: Cycling

The FITT principle can be used to develop appropriate guidelines for all three types of exercise training – aerobic, resistance, and flexibility.

RECOMMENDATIONS

OUR MOST IMPORTANT MESSAGE IS SIMPLE – MOVE MORE!

Focus on doing what you can initially, and slowly build upon this.

Did you know?

- Bouts of activity only need to be 5-10 minutes long, try breaking up activity throughout the day.
- Research has consistently shown that some physical activity is always more beneficial than nothinig (e.g., sitting or lying down for long bouts of time). Benefits are seen for all types of activity.

SAMPLE

WARM UP

- 5-10 minutes of light stretching and movement (i.e., walking/jogging on the spot, arm circles) incorporating the parts of your body being trained during the exercise session.
- Increases blood flow to the working muscles and prepares your muscles and joints for the activity.

COOL DOWN

- Follows immediately after a workout and reduces the risk of side effects caused by stopping exercise too suddenly (e.g., heart complications).
- Include exercise at a low intensity for 5-10 minutes to allow the heart rate and blood pressure to return to pre-workout levels.

MONITORING INTENSITY

The **Rating of Perceived Exertion (RPE) Scale** is often used to measure exercise intensity. RPE is measured on a scale of 0 to 10 with 0 being no exertion/effort at all, and 10 being a maximal effort.

Your goal during exercise sessions is to find an intensity that you can maintain and that feels comfortable. This number can be DIFFERENT FOR EVERYONE! Work at a pace you enjoy, and where you feel in control.

SAMPLE

**Rating of Perceived Exertion** – ***How hard are you working?***	
**0**	**No exertion** *(resting, sitting)*
**1**	**Very light** *(little or no fatigue)*
**2**	**Fairly light**
**3**	**Moderate** *(comfortable, slightly elevated breathing)*
**4**	**Somewhat hard** *(breathing deeper, light perspiration)*
**5**	**Hard** *(breathing deeply at a comfortable level, general fatigue, perspiration)*
**6**	
**7**	**Very hard** *(definite fatigue, breathing hard, heavy perspiration)*
**8**	
**9**	**Extremely hard** *(extremely vigorous, cannot maintain for long)*
**10**	**Maximal exertion**

SAFE EXERCISE GUIDELINES

1. Wear appropriate clothing
2. Warm-up
3. Use proper breathing techniques
4. Stay hydrated
5. Cool-down
6. Stretch following any exercise session
7. Rest between resistance training
8. Soreness is normal post-exercise (pain is not – listen to your body).

WHEN TO STOP EXERCISING

Being active is usually quite safe. But in rare cases, problems can arise. Be aware of some of the signs and symptoms of when to stop exercising.

Symptoms	What to Do
**Chest pain or discomfort**<br>Uncomfortable feeling of pressure, pain, squeezing or heaviness (in the center of the chest, throughout the chest, or spreading to the shoulder(s), arm(s), neck and back.	**Stop and rest**<br>If it doesn't go away after 2-4 minutes, call 911 or go to the emergency room.<br>If it does go away but returns each time you exercise, see you doctor.
**Unusual shortness of breath**	These may or may not be signs of something more serious. Talk to your parents and consult your doctor.
**Dizziness or loss of consciousness**	

SAMPLE

This content will be introduced verbally or by using a worksheet following the content provided below, which will be presented in an age-appropriate manner (e.g., use of coloring activities, journaling, etc.).

This education session discusses SMARTT goals and the difference between process and outcome goals. Different strategies for attaining goals are discussed and as an IMPACT participant, you will be given an opportunity to set your own SMARTT goals.

SAMPLE

SETTING GOALS TO MAKE A CHANGE

Goal setting is an excellent first step for motivating yourself to be physically active!

Use the S.M.A.R.T.T. goal setting criteria to help put more detail into your goal.

SMARTT Goals are:

Specific: What exactly do you want to accomplish? Where? When? Why? How much?
Measurable: Are you able to assess your progress?
Attainable: Is your goal within your reach given your current situation?
Realistic: Are you both willing and able to work towards your goal?
Timely: What is the deadline for completing your goal?
Together: Who is supporting you to be active?

Here's an example of a SMARTT goal: *"For the next 8 weeks, I will commit to walking for 15 minutes after dinner with my parents."*

Consider these points when developing your SMARTT goals:

Set both long-term and short-term goals: Set a long-term goal you want to achieve by the end of the 8-12-week exercise program and then set short-term goals; those that you want to achieve weekly that will help you reach your long-term goal.

Challenge yourself: Make your goals ambitious enough that you are proud of the accomplishment when you meet your goals.

Focus on the process: Look forward to the participation and not simply getting it over with. Goals should embrace the process that work towards the outcome.

Re-evaluate your goals regularly: Goals need to be adjusted when they've been attained – or when you are not achieving them. In order to stay motivated, set challenging goals that you can reach. Find that balance by regularly re-evaluating the goals you have set.

Outcome vs. Process Goals: Outcome goals are results that you want to achieve. These may seem unattainable so process goals are developed to help get you there. Process goals are small steps used to achieve a larger outcome goal. For example: "I want to increase my strength by increasing weight in exercises at the end of this 8-12-week program (outcome goal), and I will do this by coming to both exercise sessions each week (process goal)."

SAMPLE

MAKING YOUR GOALS WORK FOR YOU: THE RIGHT MOTIVATION

To be successful in attaining your goals, you must first find the motivation to get started and persist until your goals are reached, even in the face of obstacles. Sometimes the biggest hurdle can be getting out of your chair or bed and putting on your running shoes to go for a walk or to the gym.

Here are some tips on how to stay motivated throughout your exercise program:

Do start with small steps. Motivate yourself from the chair, to the shoes, and to the exercise (starting to exercise is the most difficult part!).

Do focus on enjoying the experience—the people, the movements, the environments, and the feelings.

Do reward yourself for a job well done. It may be as simple as occasionally enjoying an ice cream cone or taking a day off to rest and recover with movies, card games or board games.

Do surround yourself with people that are supportive of your exercise program and may be willing to exercise with you.

Do visualize success by creating an image of success in your mind. It can be a powerful tool for getting through the rough times and building motivation, self-confidence, and commitment. Take a quiet moment to close your eyes and see yourself engaged in new, more positive health habits (such as walking or playing and enjoying the weather). Picture yourself reaching your goals and enjoying the rewards of a healthier, more active lifestyle.

Do monitor your progress. When you first start making changes, you may progress rapidly. Sometimes a "plateau" or "level off" in benefits is then experienced. Use the exercise log at the end of this book to chart your progress and identify plateau or level off areas so that you can make adjustments.

Do work at a comfortable pace. Enjoy simply being active.

Do remind yourself of the benefits of being active Instead of saying "I should exercise," say "It would be better for me if I went for a walk today because I could use some fresh air".

SAMPLE

GOAL-SETTING WORKSHEET

Use this page to write down a few goals and refer to them occasionally to remind you why you're working so hard. Don't forget to make them **Specific, Measurable, Attainable, Realistic, Timely and Together.**

Goal #1:

____

____

____

Goal #2:

____

____

____

Goal #3:

____

____

____

SAMPLE

Key Point! Remember to evaluate your goals on a regular basis. Did you meet your goal? If you did, reward yourself and create a new and more challenging goal. If you didn't meet your goal, don't worry, it happens to the best of us…readjust by asking what happened – was there a barrier that needs to be addressed? How can you adapt to meet your goal?

Education Topic Three: Keeping on Track

This content will be introduced verbally or by using a worksheet following the content provided below, which will be presented in an age-appropriate manner (e.g., use of coloring activities, journaling, etc.).

This education session discusses the steps towards positive behaviour change. It also discusses ways to stay on track.

SAMPLE

STEPS TOWARD CHANGE

It is common to encounter surprises on the way to achieving a behavior change goal. Even the most well-thought-out plans usually require some adjustments. By giving yourself room to take risks, make mistakes, and apply new knowledge, you'll be able to take challenges in stride and come out stronger than ever.

Anticipate Obstacles
Occasional setbacks occur no matter how carefully you've constructed your exercise plan. Preparing for potential obstacles is an important key to overcoming them. Try listing strategies for dealing with challenges to meeting your exercise goals. If you skip a day of the program or are having difficulty reaching your goals, take time to determine how best to get back on track.

Monitor Your Progress
When you first start making changes, you may progress rapidly, noticing significant impact from engaging in regular exercise. This may then start to "level off", which is normal as your body adjusts to the exercise. Continue to monitor your progress, and reflect on how much you've achieved and how far you've come. Let your instructor know if something is too easy or too hard, and adjustments can be made!

Reward Yourself
Be patient; it will take some time before your exercise habits feel natural. Be your own cheerleader, supportive and encouraging, focusing on your achievements rather than your setbacks. Give yourself the recognition you deserve.

Visualize Success
Creating an image of success in your mind can be a powerful to build motivation, self-confidence, and commitment. Take a quiet moment to close your eyes and see yourself moving more, engaging in your exercise routine.

SAMPLE

PLANNING AHEAD to MAINTAIN YOUR HABIT

Missing your exercise sessions, not getting in as much physical activity as you planned, and moving less (not more!) are often realities. Acknowledging it will happen is important – it allows you to create strategies to deal with it to ensure you continue to move more!

Some of the most common barriers to exercise are:

Lack of Time

- The evidence is in – move more! Even 5 minute bouts of movement provide benefits…and you can add those bouts up over your day. If you can't afford large chunks of time, aim for short bouts of activity spaced comfortably throughout your day.

Lack of Wanting to Do Exercise

- The real secret is to find activities you enjoy. Do this, and you won't have to coax yourself to be active. Enjoyment = motivation to move more!

Lack of Partner

- Our program is delivered 1:1, however your parents or siblings are welcome to join in the exercise sessions – this is important to harness the social support that we know is so important for helping you “stick to an exercise habit”. Outside of session – walk with a friend, play with a friend, or socialize before or after your movement. Keep it fun by keeping it social!

SAMPLE

Lack of Ability

- Our exercise programs are tailored to you – adjusted for where you are! Always ask the instructor of a movement feels too hard or too easy. That’s the best part about exercise – any movement can be adjusted so it feels good for you!

Other potential barriers: You may have other barriers, specific to your situation, coping with your cancer, or health issues.
It is important to discuss these barriers that may prevent you for ultimately reaching your physical activity goals. Planning ahead for the ‘tough’ times may help you to stay active.

Have you ever had trouble maintaining an exercise program that you have previously started? If so, why?

If you have had difficulty previously, have certain behaviours or actions helped you to get back on track (i.e., joining a class, support from friends or parents, setting goals)?

What barriers do you anticipate will make it tough to keep up your physical activity routine? How will you handle these situations to increase your chances of being successful?

What will help you get started again if you do have a break?

SAMPLE

MAINTAINING MOTIVATION AND PERSONAL CONTROL

Motivation is an important component of continued physical activity participation. It can be useful to increase your awareness of the reasons that may motivate you to increase your physical activity level.

Research shows that the greatest sources of motivation are in doing something for:

- Fun, enjoyment, stimulation
- A feeling of accomplishment
- The pleasure of learning or mastering skills
- A benefit such as sleeping better and feeling calmer

Pursue an activity you enjoy! For a feeling of accomplishment, pursue an activity you know you can do. When you succeed, you feel competent and your motivation increases.

How do you motivate yourself to be active?

___
___
___

SAMPLE

What is NOT motivating for you in terms of physical activity?

___
___
___

In the past, how have you motivated yourself to exercise when you really didn't feel like it?

___
___
___

What health benefits motivate you to be active? Examples include: feeling better, having more energy, managing stress, feeling healthier, having more confidence, managing weight, feeling strong, looking better, sleeping better, and having fun!

___
___
___

CONTROL

Control plays an important role in our health outcomes. Although many factors in our lives are beyond our control, the actions we take in response to those factors are decidedly our own. Believing that you are in control of your own life may motivate you to build the habit of moving more.

EXERCISE YOUR WAY TO SELF-ESTEEM

Self-esteem, the view we have of our own worth, is of paramount importance to our well-being. The key to self-esteem is developing self-acceptance and feelings of competence.

To develop self-acceptance:

- Learn to accept your physical build and ability
- Focus on mastering a physical task or skill instead of comparing yourself with others

To develop feelings of competence:

- Set a simple, achievable goal for a physical activity of your choice
- Determine when you will find the time to accomplish your goal
- Have a second plan in case something happens
- Enjoy the feelings of accomplishment and competence you get as you pursue and achieve your goals

USE MOVING MORE – WHATEVER THIS LOOKS LIKE FOR YOU – TO FEEL GREAT ABOUT YOURSELF, IN CONTROL, AND COMMITTED TO WELLNESS.

SAMPLE

Session Four: Relaxation and Mindfulness

This content will be introduced verbally or by using a worksheet following the content provided below, which will be presented in an age-appropriate manner (e.g., use of coloring activities, journaling, etc.).

This education session discusses the basic concept of stress and the role of exercise in stress reduction and cancer related fatigue. It also provides information on the importance of sleep and the mind-body connection.

SAMPLE

RELAXATION

Relaxation: a state of relaxed attention to repetitive stimulus that reduces 'inner dialogue' (Benson, 1977). Relaxation counters the stress response and improves health, wellness, and quality of life.

Relaxation Benefits: (Elbe et al., 2007)
- Learn to pay attention to tension and relaxation
- Optimize and enhance recovery
- Encourage self-regulation (monitor and control one's own behaviour, emotions, and thoughts)

Relaxation Training: (McGrady, 2007):
- Relaxation training is a structured process that requires practice
- Relaxation techniques do not eliminate stress, but instead allow a child to learn to perceive situations differently and change their behavior during or after stress.
- All relaxation techniques include three components:
  - Passive attitude towards intruding thoughts
  - Repetition
  - Quiet and peaceful mind
- Examples:
  - Body scan
  - Progressive Muscle relaxation
  - Meditation
  - Yoga
  - Stretching
  - Self-hypnosis
  - Prayer
  - Music / Sound
  - Diaphragmatic breathing

SAMPLE

MINDFULNESS

Mindfulness, simply put, is an awareness of what is happening within and around you without interpretation or judgment. There are a growing number of studies showing the benefits of mindfulness for adults affected by cancer. Mindfulness has been found to reduce feelings of anxiety, stress, and depression while improving mood, cognition (e.g., memory), and well-being.

Mindfulness exercises can include breathing techniques, guided imagery, and other practices to relax the body and mind and facilitate a mind-body connection. Some examples include:

Paying attention and slowing down routines: It can be really hard to slowdown when the rest of the world is rushing by. One simple way to pay attention and slowdown is to deliberately experience an everyday task with all of your senses — touch, sound, sight, smell, and taste. For example, next time you have your favorite food, take the time to look at it, notice the colour, textures, and shape. Then begin to touch it, maybe roll it around in your fingers, hold it in the

palm of your hand. Then move on to smelling it, notice how you feel when you inhale the scent. Then gently place it in your mouth and slowly move it across your tongue. What flavours stand out to you? Choosing one task every few days can be a simple way to experience and truly enjoy it.

Finding stillness: When we get still we have the opportunity to observe thoughts, sensations, and feelings. Find a comfortable seat or lay down in a comfortable position. Set a timer for anywhere from 1-5 minutes. Close your eyes. Scan your body for sensations. Feel every inch of your body. If you need to fidget do so, but return to stillness. See if you can explore the sensations you notice and feel how they are heightened or muted. Allow your thoughts to flow. If a thought arises, let it go so it can be replaced by another. Continue until your timer goes off.

Focusing your attention: In any one moment, multiple thoughts are moving through our minds. This can result in feeling scattered, overdrawn, and overwhelmed. By focusing our attention, we can give our incredible multi-tasking brains a break and practice focusing our attention so that we can start to choose where our energy goes. Two attention focusing practices are provided below:

> **Candle exercise:** This type of *trataka* (to look or gaze) meditation involves fixing your eyes on the flame of a candle. Any candle works, from tea lights to larger candles. To prepare, choose a quiet and slightly darkened space with a safe, level surface for your candle that is free of flammable materials. Light your candle, place it on the surface, and have a seat. Allow your eyes to focus on the flame. Continue to stare at the candle and allow it to be your main focus. Try to hold your eyes steady. If you blink or get distracted, just return your attention to the flame. After 1-2 minutes, or when you feel ready, close your eyes. Focus on the 'after' image of the flame. See the flame etched in your memory. If you lose it, open your eyes gently, watch the flame and then try re-closing your eyes.

> **Body scan:** This mind-body awareness exercise involves lying in a comfortable position your back with your arms and legs extended out to the sides. Take 3 slow inhales and 3 slow exhales. Then begin to focus your attention, slowly and deliberately on each part of your body. When focusing your attention, it can be helpful to visualize a white light surrounding that body part. Start at the top of your head and move down to your toes. As you transfer your focus from body part to body part, notice any sensations, emotions or thoughts that arise. Once you notice them, accept them and move on to the next body part.

Incorporating breathwork: Often times when we are stressed or moving through our daily routines, we are not taking full inhales or exhales. Pausing and taking time to focus on our breathing is a simple way to practice mindfulness anywhere! Though there are several different types of breathwork practices, three that are great for promoting mind-body awareness and that facilitate mindfulness are below:

> **Essential breath:** This practice involves quieting the mind, stilling the body, and observing the breath. It can be done seated or lying on your back. Close your eyes or soften your gaze. Relax your hands by your sides. Without changing it, notice the quality of your breath. Are you breathing through your nose or mouth? Where in your body can you feel

SAMPLE

movement as a result of your breath? Follow your next exhale to completion, notice the slight pause, or emptiness at the bottom of your exhale. From this space allow the next inhale to arise spontaneously. Feel the expansive quality of the inhale. Continue for 5-10 breaths or longer.

Counted breath: For this type of breathing, you will inhale for a pre-determined count and exhale for a pre-determined count. For example, you can breathe in for the count of 3 and breathe out for the count of 3. You can do this for 1-2 minutes. Another example would be to breathe in for a count of 3 and breathe out for a count of 4. Continue for 5-10 breaths or longer.

Mantra breath: Mantra meditations are a great option if you have an overactive mind. Simply choose a mantra, which is a word, a phrase, or an intention that you like and repeat it to yourself as you breathe. For example, if you wish to feel calmer and more centred your mantra could be "calm, centred". As you inhale, state the word 'calm' in your mind, as you exhale state 'centred'. Continue for 5-10 breaths or longer.

Move mindfully: Movement can be a great way to practice mindfulness. Not only can it promote a greater mind-body awareness, but it can also help you practice shifting your attention from physical sensations, to your breath, to the ambient environment. Just like all things, it is best that you choose the type of movement you like most, though two common examples are provided below:

SAMPLE

Walking meditation: Next time you set out on a walk, start to walk very slowly, focusing on the experience of walking. Become aware of the sensations in your feet as you place one foot in front of the other, notice the subtle movements that keep your balance, feel the gentle sway of your arms. Continue noticing the physical sensations in your body, then begin to turn our attention outward. Begin to notice the temperature of the air on your face. Listen to the sounds closest to you and then begin to radiate your hearing outward listening for sounds further and further away. Begin to draw your awareness back to yourself and your body.

Try a yoga class: Yoga can be an excellent gateway to mindful movement. When looking for a yoga class search out words like "slow glow", "gentle flow", "yin", and be sure to ask about the instructor's credentials. Your instructor should have at least their 200-hour certification and experience modifying for adults affected by cancer or other chronic conditions. We offer a Yoga Thrive certification to yoga instructors to equip them to work with adults affected by cancer. You can contact the Health and Wellness Lab if you need a little bit of extra help finding a yoga class. Above all else, when you are practicing yoga, trust that your body knows best. If ever you feel uncomfortable in a pose, adjust, modify, or skip it!

If after reading this you feel like you are ready to incorporate some mindfulness exercises into your life, start with something you think you will like. Which of the examples listed above sounds most interesting to you? Incorporating a new behaviour, like mindfulness, should feel fun and exciting. Start off by choosing something you think you will enjoy! Next, set aside some

time when you can be in a quiet place without distraction or interruptions to practice and try out different techniques. You can start with just 1-5 minutes a few times a week. Keep track of how you feel. Notice what exercises you like and get the most benefits from.

Online resources: There are numerous online resources available. A few that we like are listed here: Headspace, Calm, Aura, Stop, Breathe & Think, InsightTimer. You can also find guided mediations available on platforms like YouTube (e.g., chair yoga) and Spotify (e.g., body scan). When looking for online resources ensure you check for credentials of the person delivering the content and that you like the sound of their voice. Though the latter might seem insignificant it will be important to help you experience deeper sense of mindfulness!

MIND-BODY CONNECTION

Your body responds to the way you think, feel and act. This is often called the "mind-body connection." When you are stressed, anxious, or upset, your body tries to tell you that something isn't right. To help effectively manage stress, anxiety, depression, and pain, as well as some of the side effects of treatment, many survivors often use imagery and relaxation techniques.

What stress management techniques do you currently use? What techniques have you used in the past?

____
____
____
____

Have you ever tried imagery or relaxation? Would you consider trying a new method?

____
____
____
____

SAMPLE

Session Five: Social Support and Long-Term Exercise Maintenance

This content will be introduced verbally or by using a worksheet following the content provided below, which will be presented in an age-appropriate manner (e.g., use of coloring activities, journaling, etc.).

This topic discusses social support and where people can identify different sources of social support for maintaining exercise. Tips for maintaining long-term physical activity beyond this program are also provided.

SAMPLE

SOCIAL SUPPORT

Social support is an important ingredient in the exercise behaviour change process. You are more likely to be successful if your parents, siblings, and friends are supportive of your efforts toward building an exercise, or movement, habit. Social support can occur in many forms. It may be nothing more than encouragement and reinforcement of your attempts to change your exercise habits, or it may actually include engaging in physical activity with you!

WHO is supporting you??
Likely sources of support may include **Parents**, **Grandparents**, **Friends, Neighbors**, **or Teachers.**

YOUR SUPPORT SYSTEM

Who do you feel is providing you with social support?

____

____

____

____

What kinds of social support do they provide to help you make your lifestyle change?

____

____

____

____

How could you improve your support system?

____

____

____

____

SAMPLE

TIPS TO ENCOURAGE PHYSICAL ACTIVITY MAINTENANCE

To continue to experience benefits from physical activity, you need to continue to maintain your physical activity habits. Here are some tips for long-term maintenance!

1. **Continue to Look at Exercise Differently**
   All movement is exercise. Take a dog for a walk, bike to school, take five-minute stretch breaks. REMEMBER OUR SIMPLE MESSAGE…MOVE MORE!

2. **Think Small**
   Even a 5-10 minute brisk walk at the end of the day is beneficial…small bouts throughout the day may help with your energy levels. Move even a little bit for benefits!

3. **Set Goals, Plan Your Weekly Activity**
   Set a weekly goal, such as increasing the speed, duration, or other aspect of your activity…then see where in your daily movement you can build in progress towards reaching your weekly goal!

4. **Get Off the Beaten Path**
   Check out our resource package for other movement options…if you continue with an exercise class, that is great! But there are lots of options out there. Pick what you'll enjoy doing. Remember – ENJOYMENT = MOTIVATION!

5. **Use Your Brain**
   As you move more, focusing on your breath or concentrating on the movement of your body, can help you get more out of the exercise.

6. **Get an Accountability Partner**
   Find a friend, family member, fitness partner or leader to keep you honest. You can either exercise with your partner, or simply check in to report your progress.

7. **Plan to Stay Active**
   Plan ahead, to build more movement into your daily activities. Schedule it. Then follow through. Plan to walk to school and put your walking shoes at your front door the night before. Plan to take that new yoga class next week.

8. **Address Your Barriers**
   Make a plan and stay motivated to move more.

9. **Go Group**
   Even if you are introverted, the presence of others in your exercise environment can be motivating. Encourage your family and friends to join into the exercise sessions with you!

SAMPLE

10. Use a Script to Stick with Your Exercise Habit

We tell ourselves things like, “skipping this one little walk won’t matter all that much”. Be prepared with an answer for this excuse. Use images of past successful experiences to remind yourself of how good exercise makes you feel. Or repeat a simple phrase to yourself, such as: “Every little bit makes a big difference”. If you use planning, flexibility and imagination, you will be successful.

TAKE HOME MESSAGE

Pursue activities that you enjoy! For a feeling of accomplishment, pursue an activity you know you can do. When you succeed, you feel competent and your motivation increases. If you enjoy learning, try a new activity or build new skills. Whatever benefits you seek — companionship, a feeling of well-being, fitness improvements, health — match them with a physical activity than can provide these benefits.

Above all, make it convenient to be active. Many activities are accessible right from your front door (i.e., walking, playing, and biking). Take every opportunity to be active…try spacing a few short bouts throughout the day. Get yourself into a routine and the benefits will start accumulating.

SAMPLE

MOVE MORE!

Being mindful of the healthy lifestyle you have worked to establish and want to maintain is part of ensuring that you will continue to live in a healthy and balanced way. Consider the following questions as you move through this program and beyond:

How do you plan to continue exercising on a regular basis?

Where, when, and what exercise do you plan on doing?

How has participation in this program impacted your life or quality of life?

SAMPLE

What specific benefits of being physically active have you enjoyed the most?
