# Supplementary material for: IMplementation of Physical Activity for Children and adolescents on Treatment (IMPACT) for Cancer Diagnoses in Alberta: Protocol for a Single-Arm, Mixed-Methods, Hybrid Effectiveness-Implementation Trial
Source: JMIR Res Protoc. 2025 Dec 17;14:e59302. doi: 10.2196/59302 (PMC12756665; doi:10.2196/59302)
Supplement: Multimedia Appendix 3 [file resprot_v14i1e59302_app3.pdf]

Supplementary File 3. Semi-structured interview guides

[CHILDREN (5-10)]

Thank you for your involvement in the IMPACT program. We want to make this program available to more children like you. We also want to make sure that people like the program and find it good. Today, we will talk about the moving, playing, and the IMPACT program. We will also talk about what you liked or did not like. There are no right or wrong answers, and all of your thoughts are important to us.

1. Let's begin by talking about moving and playing. Can you tell me why you move/play?
   PROBE: Can you tell me about your favourite ways to move/play? Least favourite?

2. Now, let's talk about the IMPACT program, can you please explain the program to me?
   Follow-up questions to be posed based on ideas presented by participant.

3. What did you like/dislike about the IMPACT program?
   Follow-up questions to be posed based on ideas presented by participant.

4. What things made it easier/harder to participate in the IMPACT program?
   Follow-up questions to be posed based on ideas presented by participant.

5. How do you feel when you move more? Do you feel happy? Do you feel tired?
   Follow-up questions to be posed based on ideas presented by participant.

6. Is there anything else you'd like to tell me about the IMPACT program or moving and playing?

7. Is there anything else you'd like to add?

[TEENAGERS/ADOLESCENTS (11-18)]

Thank you for your involvement in the IMPACT physical activity program. We want to make this program available to more children and adolescents like you. We also want to make sure that people like the program and find it helpful. Today, we will talk about moving, physical activity, and the IMPACT program. We will also talk about liked or did not like, and any changes you may have noticed in the way you feel. There are no right or wrong answers, and all of your thoughts are important to us.

1. Let's begin by talking about movement and movement and physical activity. Can you tell me why you move and do physical activity?

PROBE: Can you tell me about your favourite ways to move/play? Least favourite?
Follow-up questions to be posed based on ideas presented by participant.

2. Now, let's talk about the IMPACT program. In your own words, can you please describe the program to me? How did you hear about the program?

PROBE: Can you describe what this experience has been like for you? When you first started what did you expect?

Follow-up questions to be posed based on ideas presented by participant.

3. What were your favourite/least favourite parts of the IMPACT program?

PROBE: What could you change, if anything, to improve the program?
Follow-up questions to be posed based on ideas presented by participant.

4. What things made it easier/harder for you to participate in the IMPACT program?

Follow-up questions to be posed based on ideas presented by participant.

5. I am going to ask you to try to remember a few things. If you cannot that is OK. Can you tell me how you felt before/after each IMPACT session? How did you feel at the end of the IMPACT program compared to when you started?

Follow-up questions to be posed based on ideas presented by participant.

6. Were you (are you) physically active outside of your time in the IMPACT physical activity program?

Follow-up questions to be posed based on ideas presented by participant.

7. Is there anything else you'd like to tell me about the IMPACT program, moving or physical activity?

8. Is there anything else you'd like to add?

[PARENTS/CAREGIVERS]

Thank you for supporting your child's involvement in the IMPACT program. We want to make this program available to more children like yours. We also want to make sure that people like the program and find it helpful. Today, we will talk to you about your child's movement, physical activity, and participation in the IMPACT program. We will also talk about what you and your child liked or did not like, and any changes you may or may not have noticed in your child(ren). There are no right or wrong answers, and all of your thoughts are important to us.

1. Can you tell me about your child's movement and physical activity? Before IMPACT/During IMPACT/After IMPACT?

    Follow-up questions to be posed based on ideas presented by participant.

2. What were your thoughts on the referral process for your child to join the IMPACT program?

    PROBE: [Parents of minors]: What were you reasons for deciding to allow your child to participate in the IMPACT program? [Parents of mature minors]: What were the reasons you wanted your child to participate in the IMPACT program?

    Follow-up questions to be posed based on ideas presented by participant.

3. When your child first started, was there anything that you expected they would gain? How did/didn't your expectations change?

    Follow-up questions to be posed based on ideas presented by participant.

4. What was your experience supervising and/or participating with your child within the IMPACT program like? What has made it (would have made it) easier for you to help your child participate/attend the IMPACT program? To feel engaged in the sessions?

    Follow-up questions to be posed based on ideas presented by participant.

5. What elements of the IMPACT program did you useful/beneficial for you and your child? What did you like/dislike?

    Follow-up questions to be posed based on ideas presented by participant.

6. I am going ask you now in general, how did your child feel before/after each IMPACT session? What changes, if any, did you notice in your child over the course of the IMPACT program?

7. What did you think about the physical activity program being delivered online (Zoom) while your child was in the hospital/at home?

    Follow-up questions to be posed based on ideas presented by participant.

8. Is there anything else you'd like to tell me about the IMPACT program, moving or physical activity?

9. Is there anything else you'd like to add?

[Healthcare providers from Stollery Children's Hospital and Alberta Children's Hospital]

Thank you for your support of the IMplementation of Physical Activity for Children and adolescents on Treatment or IMPACT intervention. As a reminder, the IMPACT intervention is a 1:1 physical activity intervention delivered to children/adolescents affected by cancer and/or blood disease who are pre-treatment, on treatment, or within 3 months of treatment. The physical activity sessions are tailored and delivered online over Zoom for 12 weeks.

Today, we will talk to your experience, if any, with the IMPACT intervention. There are no right or wrong answers, and all of your thoughts are important to us.

Do you have any questions, before we begin? [IF YES, ADDRESS. IF NO, PROCEED TO QUESTIONS].

OK, I will start the recorder now.

1. Please tell me about your role at [INSERT HOSPITAL NAME], your current title, your training, and how many years you've been in your role. If you're comfortable, please share your pronouns and biological sex.

2. Had you heard of the intervention prior to this interview? Can you please explain your involvement with the IMPACT intervention? What experience, if any, have you had with it?
    PROBE: Tell me more.

3. Did you refer to the IMPACT intervention? If yes, why? If no, why not? Do you think you referred more/less than your colleagues?

4. What would have made it easier for you to refer?
    PROBE: Tell me more. Why do you think that? Do you have any ideas about how to make this easier?

5. What things made it harder for you to refer?
    PROBE: Tell me more. Why do you think that? Do you have any ideas about how to make this easier?

6. What do you think your colleagues think of the IMPACT intervention? Of referring?
    PROBE: Tell me more. Why do you think that? What have they said?

7. Have you heard about the IMPACT intervention from your patients? What did they say? Have you heard about the IMPACT intervention from your colleagues? What did they say?
    PROBE: Tell me more. Why do you think that? What have they said?

8. What do you think about the physical activity intervention being delivered online (Zoom) while your patient was in the hospital/at home?
    PROBE: Tell me more. Why do you think that?

9. Is there anything else you'd like to tell me about the IMPACT intervention so far? Is there anything else you'd like to add?

Qualitative Interview Guide

[Physical activity specialists involved in delivering the IMPACT intervention or conducting physical assessments]

Thank you for your support of and involvement with the IMplementation of Physical Activity for Children and adolescents on Treatment or IMPACT intervention. As you know, we want to make this intervention available to more children and adolescents affected by cancer and/or blood disease, and to do that will need to train and support more qualified exercise professionals to deliver this intervention, and conduct the physical assessments. There are no right or wrong answers, and all of your thoughts are important to us.

Do you have any questions, before we begin? [IF YES, ADDRESS. IF NO, PROCEED TO QUESTIONS].

OK, I will start the recorder now.

1. Please tell me about your current title, your training, and how many years you've been in your role. If you're comfortable, please share your pronouns and biological sex.

2. What training did you have prior to starting with the IMPACT intervention (e.g., CPT, CEP, Thrive Health)?

    PROBE: How was this training helped you with delivery IMPACT sessions?

3. Beyond your previous training, what did you think about the IMPACT-specific training you received (e.g., information sessions from content experts, scenario-based training, PEER shadowing, practicing with healthy children)?

    PROBE: Tell me more. Why do you think that?

4. Did you feel equipped to [plan physical activity sessions OR conduct physical assessments] for children and adolescents affected by cancer or blood disease? what were your concerns? Did you feel equipped to deliver physical activity for children and adolescents affected by cancer or blood disease [only for PA specialists]? What were your concerns?

    PROBE: Tell me more. Why do you think that?

5. What additional information or support would you have liked access to prior to starting with the IMPACT intervention? During [delivery OR assessments]?

    PROBE: Tell me more. Why do you think that? Other examples: training, practice (specific with kids), more information about the participant?

6. What has been your experience [delivering physical activity OR conducting physical assessments] online for children and adolescents affected by cancer or blood disease?

    PROBE: Tell me more. Why do you think that? What are things you enjoyed or did not enjoy?

7. What has been the hardest part?

PROBE: Tell me more. Why do you think that? Examples: communication, information you received, recording/tracking information. Do you have any ideas about how to make this easier?

8. What has made it easier for you?

PROBE: Tell me more. Why do you think that? Do you have any ideas about how to make this easier?

9. For PA specialists: Once a child has been medically cleared and they have consented to participate in the study, the study coordinator connects with you to connect you with the participant and their caregiver. What are your thoughts on this process? For physical assessors: Once a child has been medically cleared and they have consented to participate in the study, the study coordinator connects with you and the other assessor to get your availability and then sets up a time with the participant and their caregiver for the assessment. What are your thoughts on this process?

PROBE: What could we do to make this process easier? Do you have any concerns with this process?

10. Is there anything else you'd like to tell me about the IMPACT intervention so far? Is there anything else you'd like to add?
